# Terbium radionuclides for theranostic applications in nuclear medicine: from atom to bedside

**DOI:** 10.7150/thno.92775

**Published:** 2024-02-17

**Authors:** Camille Van Laere, Michel Koole, Christophe M. Deroose, Michiel Van de Voorde, Kristof Baete, Thomas E. Cocolios, Charlotte Duchemin, Maarten Ooms, Frederik Cleeren

**Affiliations:** 1 Belgian Nuclear Research Centre (SCK CEN), Institute for Nuclear Medical Applications, Mol, Belgium.; 2 Radiopharmaceutical Research, Department of Pharmacy and Pharmacology, KU Leuven, Leuven, Belgium.; 3 Nuclear Medicine, University Hospitals Leuven, Belgium.; 4 Nuclear Medicine & Molecular Imaging, Department of Imaging and Pathology, KU Leuven, Leuven, Belgium.; 5 KU Leuven, Institute for Nuclear and Radiation Physics, Department of Physics and Astronomy, Leuven, Belgium.; 6 CERN, Geneva, Switzerland.

**Keywords:** Terbium radionuclides, Theranostics, Nuclear Medicine, Radionuclide Therapy, Molecular Imaging

## Abstract

Terbium features four clinically interesting radionuclides for application in nuclear medicine: terbium-149, terbium-152, terbium-155, and terbium-161. Their identical chemical properties enable the synthesis of radiopharmaceuticals with the same pharmacokinetic character, while their distinctive decay characteristics make them valuable for both imaging and therapeutic applications. In particular, terbium-152 and terbium-155 are useful candidates for positron emission tomography (PET) and single photon emission computed tomography (SPECT) imaging, respectively; whereas terbium-149 and terbium-161 find application in α- and β^-^-/Auger electron therapy, respectively. This unique characteristic makes the terbium family ideal for the “matched-pair” principle of theranostics. In this review, the advantages and challenges of terbium-based radiopharmaceuticals are discussed, covering the entire chain from radionuclide production to bedside administration. It elaborates on the fundamental properties of terbium, the production routes of the four interesting radionuclides and gives an overview of the available bifunctional chelators. Finally, we discuss the preclinical and clinical studies as well as the prospects of this promising development in nuclear medicine.

## 1. Introduction

Theranostics is an emerging field in nuclear medicine allowing for more personalized treatments. The term 'theranostics' refers to the combined application of a radiopharmaceutical for therapy (i.e. radionuclide therapy) and diagnostics (i.e. molecular imaging). Its profound impact lies in the ability to refine patient selection and predict treatment response and toxicity, with the aim of enhancing the therapeutic effect while minimizing treatment-related toxicities. The radiopharmaceutical usually comprises three components: (i) a radionuclide for imaging or therapeutic applications, (ii) a vector molecule that delivers the radionuclide to the cells of interest expressing a specific molecular target, and (iii) a chelator which forms a stable chemical connection between the radionuclide and the vector molecule. Importantly, the vector molecule, which can be a small (in)organic molecule, peptide or protein, must retain its affinity and selectivity for the target after conjugation with the radionuclide.

**Figure 1:**
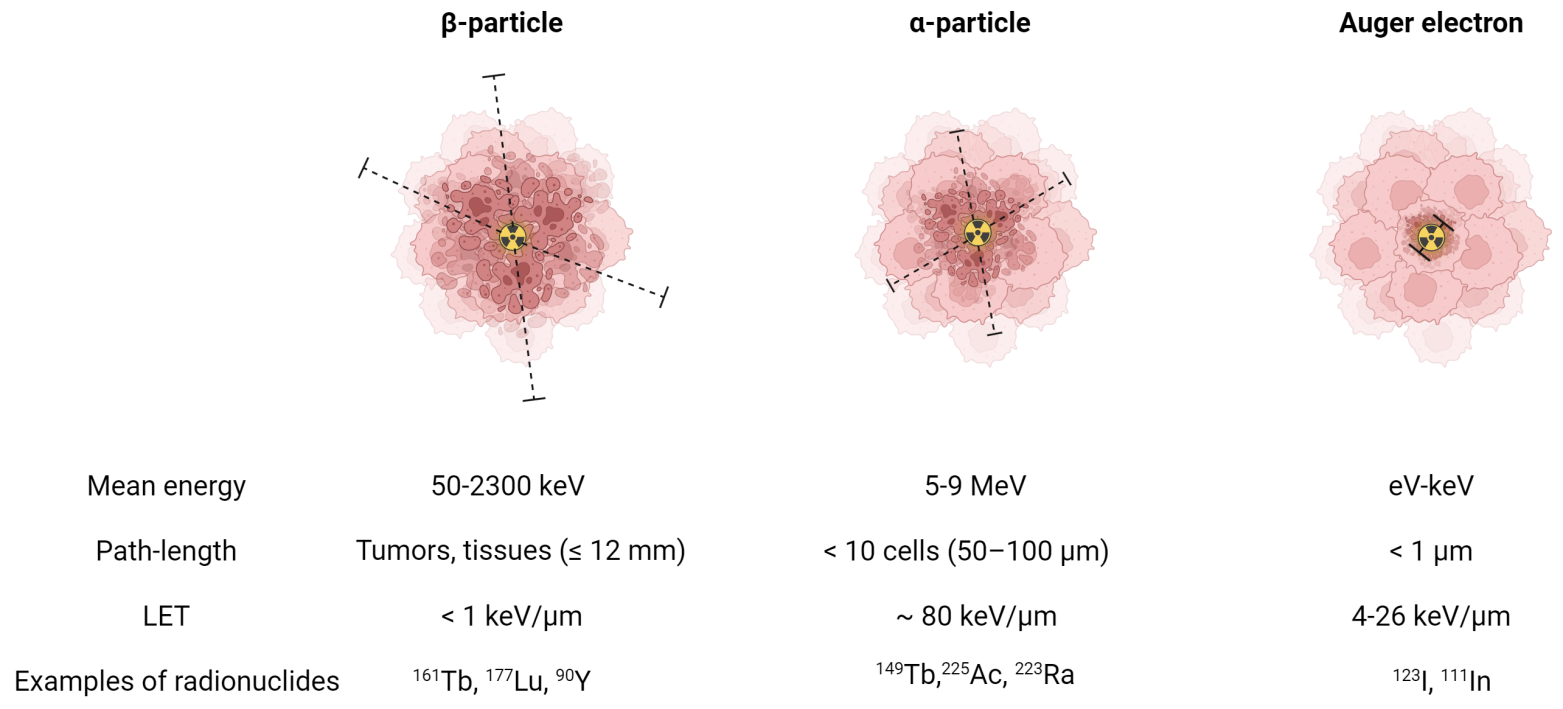
Biophysical properties of a β-particle, α-particle, and Auger electron. Dashed lines indicate the particle range on the scale of cells. (LET = linear energy transfer). Created with BioRender.com.

The suitability of a radionuclide for diagnostic or therapeutic applications is primarily determined by its decay properties 1. Diagnostic radionuclides decay by emitting electromagnetic radiation (x- and γ-rays) or by positrons which undergo annihilation upon the interaction with an electron, resulting in the emission of two co-linear 511 keV photons. These diagnostic radionuclides can be used for single photon emission computed tomography (SPECT) and positron emission tomography (PET), respectively. On the other hand, therapeutic radionuclides decay by emitting α- or β^-^-particles and/or internal conversion (IC) and Auger electrons (**Figure 1**). These forms of ionizing radiation can damage cells via three different mechanisms: direct damage, indirect damage, and the bystander effect. Direct damage occurs through the direct interaction of ionizing radiation with the DNA, causing single-stranded or double-stranded breaks. This type of damage is predominant with radionuclides with a high linear energy transfer (LET), leading to a high number of ionizations per unit path-length. In contrast, indirect damage results from the hydrolysis of water molecules, producing reactive oxygen species (ROS) which can subsequently damage essential biomolecules such as phospholipids, proteins, RNA, and DNA. Finally, radiation-induced bystander effect describes the phenomenon where cells not exposed by radiation still experience toxic effects due to signals produced by irradiated cells 1.

α-particles are characterized by a high energy (4-9 MeV) and high LET (~80 keV/μm) 2. This translates to a high rate of double-stranded DNA breaks and consequently genotoxic and irreparable damage, even in hypoxic conditions. As a result, it induces effective cell killing while being less sensitive to treatment resistance. With a moderate path length (50-100 μm), α-particles have a theoretical advantage for eliminating small tumors and micro-metastases with minimal healthy tissue exposure. In comparison, β^-^-particles are characterized by a medium to high mean energy (0.05-2.3 MeV) and low LET (<1 keV/μm). Their longer path-length (≤12 mm), depending on their energy, makes them more suitable for heterogeneous tumors with poor target expression or hypoperfusion. Auger electrons, on the other hand, are very-low energy electrons (eV-keV) with a high LET (4-26 keV/μm) and a limited path-length (<1 μm), rendering them ideal for targeting isolated cancer cells and micro-metastasis 2. Due to their limited range, Auger electrons need to be in close proximity to radio-sensitive cellular compartments to exert their therapeutic effects. Consequently, the highest relative biological effectiveness (RBE) of Auger electron emitters is expected when these radionuclides are localized within the nucleus. However, research has indicated that the cell membrane and mitochondria might also be good targets 3-5. Most Auger electron emitting radionuclides also emit a small number of IC electrons, with higher energies and ranges up to several millimeters 6. This broader range allows IC electrons to travel further distances and affect a larger volume of cells and tissue compared to Auger electrons.

In the last decade, terbium has emerged as a promising element for nuclear medicine, featuring radionuclides covering all four modalities in the field: terbium-149, terbium-152, terbium-155, and terbium-161. This unique characteristic makes terbium ideal for the “matched-pair” principle of theranostics, allowing the formation of diagnostic and therapeutic radiopharmaceuticals with identical chemical structure and pharmacokinetics. In this context, terbium-149 decays by low energy α-decay and has been proposed as a promising radionuclide for α-therapy. Additionally, the partial positron decay of terbium-149 also allows PET imaging for post-treatment assessments, enabling the visualization of the therapeutic radiopharmaceutical's distribution. Terbium-152 and terbium-155 can be used for PET and SPECT imaging, respectively. Similar to the clinically-used lutetium-177, terbium-161 decays by β^-^-emission and emits γ-radiation suitable for SPECT imaging purposes. However, terbium-161 is believed to be a more superior alternative to lutetium-177 due to the co-emission of a significant number of low-energy IC and Auger electrons which lead to a high local absorbed dose, even in micro-metastasis. Aside from the physical decay properties of the radionuclide, their application potential is also dependent on the ability to produce the radionuclides in sufficient quantities. In this regard, terbium-161 and terbium-155 are the most promising radionuclides to be made available in large quantities, potentiating their translation to the clinic.

In this review, we report on the chemical and physical properties of all four terbium radionuclides and their production routes. In addition, an overview of available bifunctional chelating agents is provided. Finally, we summarize the pre-clinical and first clinical proof-of-concept studies involving terbium-based radiopharmaceuticals and discuss the challenges and potential of these radionuclides for future use in nuclear medicine.

## 2. Radionuclide Properties and Production Methods of the Four Clinically Interesting Terbium Radionuclides

### 2.1. Decay Properties

The decay characteristics and applications of the four clinically interesting terbium radionuclides are provided in **Table 1**.

#### 2.1.1. Terbium-149

**Figure 2:**
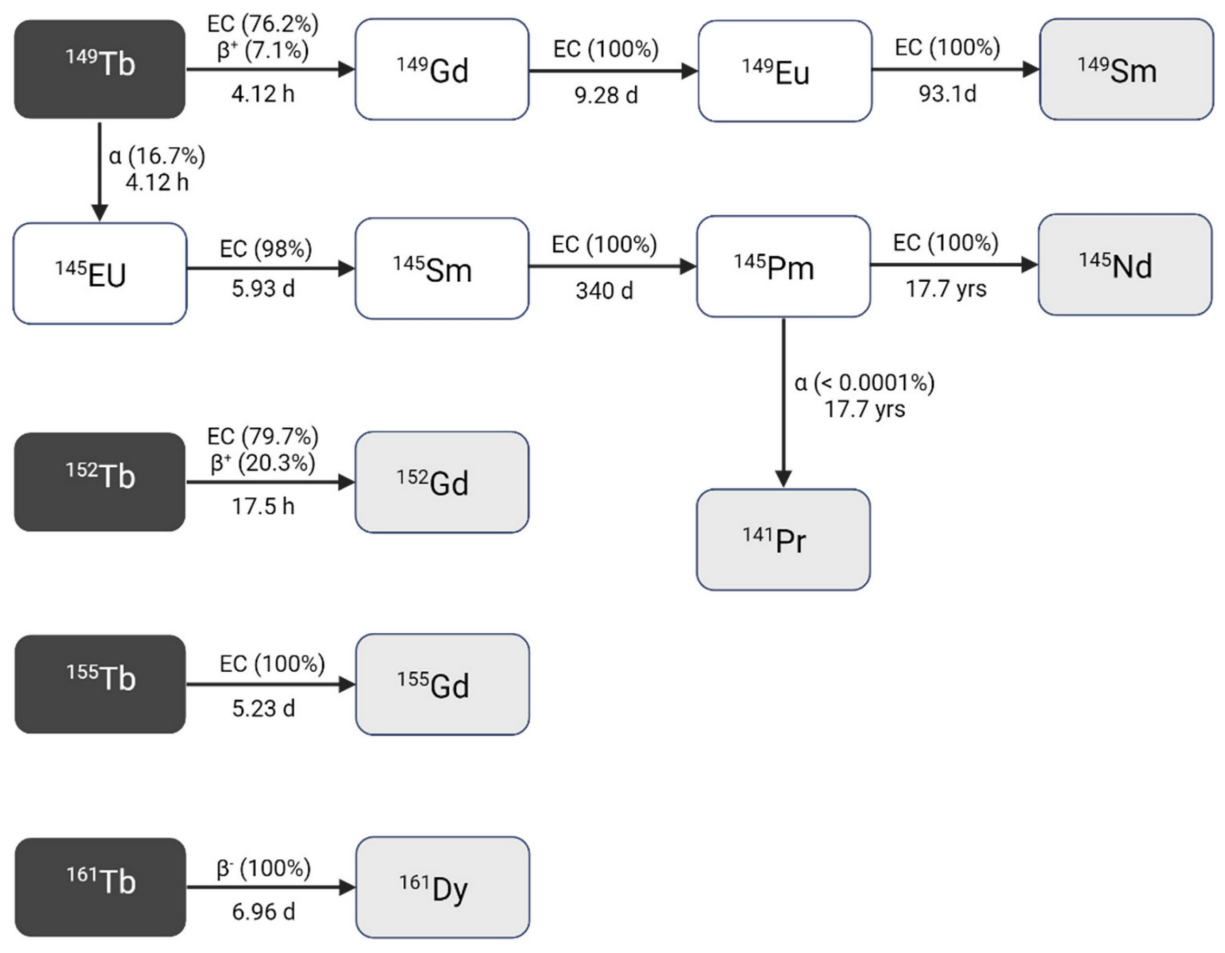
Decay scheme of terbium radionuclides 9. Created with BioRender.com.

Terbium-149 (T_1/2_ = 4.12 h) decays to several longer-lived radiolanthanides following a rather complex decay scheme (**Figure 2, Table 1**) 9,10. Terbium-149 primarily decays by electron capture (EC, I = 76.2%) and β^+^-emission (E_β+,av_ = 0.73 MeV, I_β+_ = 7.1%) to gadolinium-149 (T_1/2_ = 9.28 days). The latter subsequently decays via europium-149 (T_1/2_ = 93.1 days) to stable samarium-149. The residual 16.7% of terbium-149 decays by low energy α-decay (E_α_ = 3.97 MeV, I_α_ = 16.7%) generating an isobaric decay chain via europium-145 (T_1/2_ = 5.93 days), samarium-145 (T_1/2_ = 340 days), and promethium-145 (T_1/2_ = 17.7 years). The latter ultimately decays to stable neodymium-145 and praseodymium-141 9,10.

The α-particles emitted by terbium-149 are characterized by a short path length (25-28 μm) and very high LET (140-142 keV/μm) 11. These physical properties signify the potential of terbium-149 as a potent radionuclide for α-therapy. Furthermore, the absence of α-emitting daughter isotopes is generally regarded as an additional benefit as it prevents damage to healthy tissue by recoiling daughters. Nonetheless, the clinical implications and waste management of the long-lived daughter isotopes associated with terbium-149 remain to be determined. In addition to its therapeutic potential, terbium-149 may also be used for imaging purposes due to the co-emission of β^+^ radiation as demonstrated in a preclinical study by Müller and colleagues 11. In the context of post-treatment imaging, terbium-149 may facilitate the visualization of the radiopharmaceutical's biodistribution up to 24 h p.i. Albeit, it should be noted that the relatively low positron branching ratio (I_β+_ = 7.1%), combined with α-emission, may result in inferior image quality in a clinical setting unless high sensitivity PET cameras (e.g. long-axial field-of-view PET) or long acquisition times are used.

#### 2.1.2. Terbium-152

Terbium-152 decays with a half-life of 17.5 h to stable gadolinium-152 by β^+^-decay (E_β+,av_ = 1.140 MeV, I = 20.3%) and EC (I = 79.7%), making it suitable for PET imaging 9. However, the high energy and low intensity of the emitted β^+^-particles (E_β,av_ = 1.140 MeV, I = 20.3%) may adversely affect the image quality and should be taken into consideration. The co-emission of several γ-rays (**Table 1**) makes terbium-152 also a potential candidate for SPECT, though in nuclear medicine PET is generally preferred over SPECT due to the enhanced sensitivity and resolution it offers 9. Importantly, the SPECT images might also be compromised by the highly penetrant annihilation photons.

#### 2.1.3. Terbium-155

Terbium-155 has a half-life of 5.23 days and decays through EC to stable gadolinium-155 7,9. The emitted low energy γ-rays are characterized by three distinct energies (**Table 1**) which are ideal for low-dose SPECT imaging 9,12. Notably, terbium-155 stands out as the only radiolanthanide with suitable γ-rays for SPECT without β^+^/β^-^-emission. Consequently, terbium-155 can be considered an ideal diagnostic match to therapeutic radionuclides such as terbium-149 and terbium-161. Furthermore, its relatively long half-live makes it possible to study the biodistribution of radiopharmaceuticals over several days 12.

#### 2.1.4. Terbium-161

Terbium-161 is a highly promising radionuclide for β^-^-therapy as it decays by β^-^-emission (E_β-,av_ = 154 keV) to dysprosium-161 with a half-life of 6.96 days (**Table 1**) 8. Furthermore, the simultaneous emission of γ-rays at 48.9 keV (I_γ_ =17.0%) and 74.6 keV (I_γ_ = 10.3%) makes terbium-161 suitable for SPECT imaging 9. In fact, its decay characteristics closely resemble those of lutetium-177 (T_1/2_ = 6.65 days, E_β-,av_ = 134 keV; E_γ_ = 113 keV (I = 6.17%), E_γ_ = 208 keV (I = 10.36%)), positioning terbium-161 as a viable alternative. Additionally, the co-emission of a considerable amount of IC and Auger electrons might further improve the therapeutic potential of terbium-161 (~ 12.12 e-, ≤ 40 keV per decay) over lutetium-177 (~ 1.11 e-, 1.0 keV per decay) as demonstrated by the comparative studies performed by Müller and colleagues 13-15. Moreover, dosimetric analysis using Monte-Carlo simulations revealed that the low-energy electrons of terbium-161 effectively deliver a high local absorbed dose, even in micro-metastasis that remain undetected by pre-therapy imaging and that, if underdosed, could lead to post-therapy progression 16. The absorbed dose theoretically required to achieve metastatic control was found to be 40% lower with terbium-161 than with lutetium-177. In accordance, additional studies have shown that the cellular absorbed dose of terbium-161 is more than 3-fold higher than lutetium-177, underlining the potential therapeutic superiority of terbium-161 over lutetium-177 17-19. This advantage of terbium-161 can be attributed to the high emission of electrons with energies below 40 keV 16. Of note, this ratio decreases with increasing cell cluster size and similar absorbed doses were detected for macroscopic cell clusters of approximately 2 mm (10^6^-10^7^ tumor cells).

It is worth mentioning that compared to lutetium-177, the energies of the gammas emitted by terbium-161 are much lower, with the 75 keV photopeak being the most suitable peak for imaging 20. Initial developments in quantitative ^161^Tb-SPECT indicate its feasibility, particularly when utilizing a low-energy collimator where the higher attenuation of 75 keV photons can be effectively compensated by enhanced detection sensitivity 20. Nevertheless, addressing scatter correction may pose challenging, though the utilization of cadmium zinc telluride (CZT)-based SPECT systems could prove beneficial 21. An in-depth comparison between ^177^Lu- and ^161^Tb-SPECT has not been performed to date.

### 2.2. Current Strategies for Production

The production of adequate amounts of carrier-free terbium radionuclides constitutes a major challenge for their use in nuclear medicine. Whereas terbium-161 can be produced in nuclear reactors, the other three radionuclides can only be produced in cyclotrons or high-energy ion beam facilities. Currently, proton-induced spallation combined with on- or off-line mass separation is the optimal method for the production of terbium-149, terbium-152, and terbium-155 with sufficient quantity and purity. To date, only a few facilities are operational worldwide that allow for production of research amounts of these terbium isotopes, including ISOLDE and MEDICIS at CERN (Switzerland) and ISAC at TRIUMF (Canada) 22,23. It is important to note that the limited number of facilities that are operational to date will not be able to sustain the growing global demand for accelerator-produced terbium radionuclides. To address this challenge, new facilities are being considered that could upscale this production route, including IMPACT-TATTOOS or ISOL@MYRRHA which will operate at lower energy than CERN, namely 590 MeV at IMPACT-TATTOOS and 600 MeV at ISOL@MYRRHA compared to 1.4 GeV at CERN.

In the following paragraphs, a brief overview of the explored production methods per radionuclide is provided. Before thoroughly examining these methods, it is essential to understand the significant factors that influence the production yield and quality of the obtained radionuclide, whether produced by accelerators or reactors. These factors include the incident particle flux, reaction cross section, and isotopic and chemical purity of the target. Additionally, the duration of target irradiation and the radioactive decay constant of the produced radionuclides are important parameters. In the case of accelerators, one must also factor in target thickness, target density, and target stopping power.

#### 2.2.1. Terbium-149

The production of terbium-149 remains the most challenging, hindering its availability. For preclinical research, terbium-149 has primarily been produced by high energy proton induced spallation of tantalum targets followed by mass separation 11,24. In short, tantalum foils are irradiated with a 1.4 GeV proton beam after which the generated radiolanthanides are released from the target, ionized, and accelerated as a radioactive ion beam (RIB) and subsequently implanted on a thin layer of aluminum, zinc or NaCl. In on-line facilities, where the beam impinges on the target simultaneously with the extraction and implantation of radionuclides, terbium-149 is also produced indirectly at the end of irradiation through the decay of dysprosium-149 (T_1/2_ = 4 min) which is also implanted on the support foil. One disadvantage of this method is the potential contamination by pseudo-isobars in the final product, such as cerium-133m oxides. To obtain high radionuclidic purity, the use of cation exchange chromatography is crucial to further isolate terbium-149 from its daughter isotopes and pseudo-isobars 24. To date, production yields ranging from few hundreds of MBq up to 1 GBq have been achieved at the ISOLDE facility, though access remains limited to a few times a year due to the competitive nature of the beam allocation at that facility. The future ISOL@MYRRHA facility at SCK CEN will feature a lower primary beam energy (600 MeV), where the co-production of cerium will be suppressed 20, 32. The expected 250-fold higher beam intensity will also substantially increase the production yield. Similarly, IMPACT-TATTOOS will operate at 590 MeV with a 50-fold intensity increase compared to CERN.

**Figure 3:**
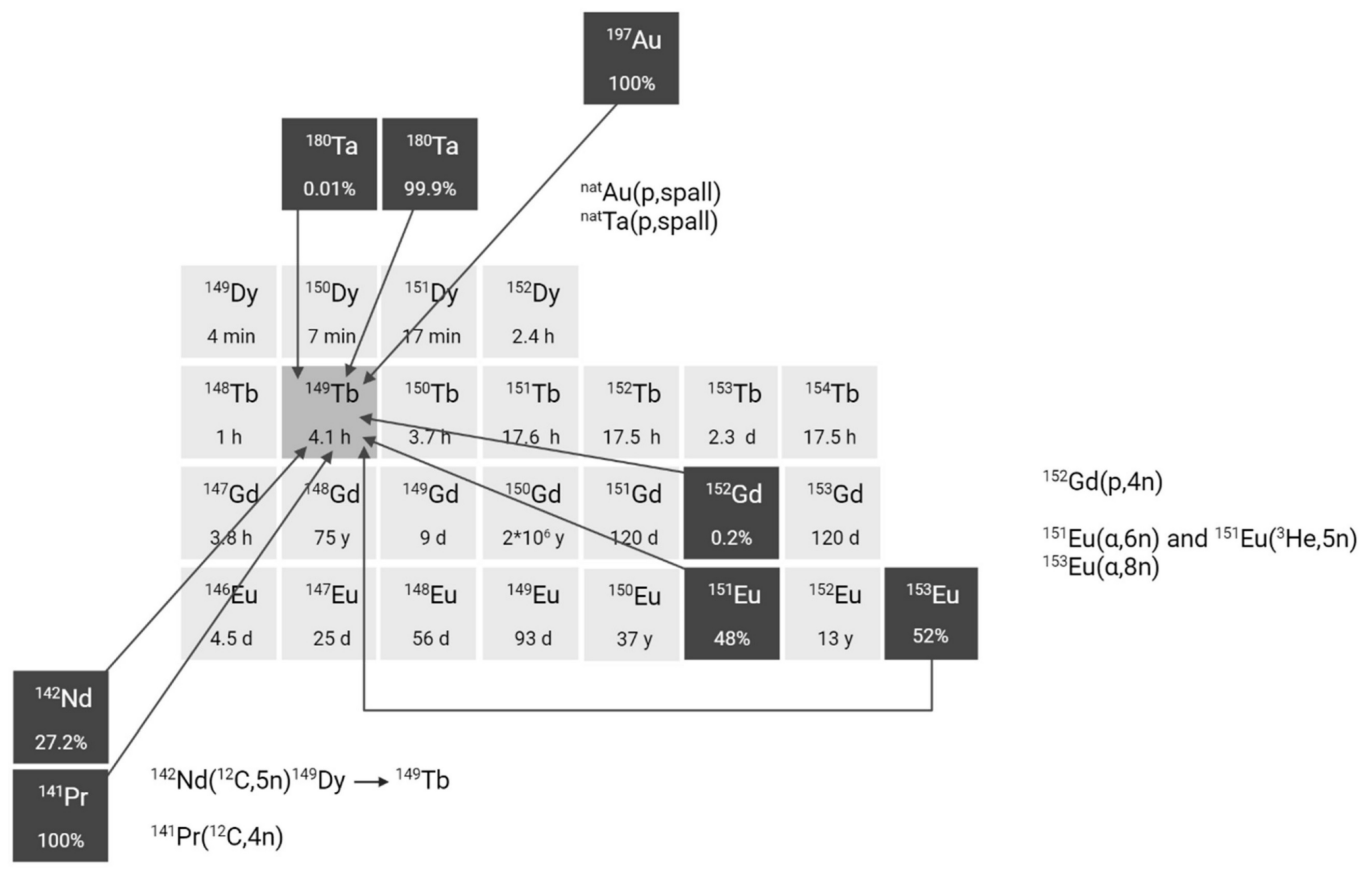
Section of the nuclide chart illustrating possible production routes of terbium-149 26. Created with BioRender.com.

Several alternative production methods via light (α or p) and heavy particle (e.g. ^12^C) induced nuclear reactions have also been investigated (**Figure 3**). Though the practical application of these approaches is hindered by the reliance on scarce, powerful accelerators and the formation of significant amounts of relatively long-lived terbium-150 and terbium-151 during irradiation Additionally, heavy ion induced nuclear reactions also lead to the generation of excited state terbium-149m (T_1/2_ = 4.16 min) which decays via β^+^ to gadolinium-149 (T_1/2_ = 9.28 days) instead of the ground-state terbium-149 25. Consequently, gadolinium-149 needs to be removed using chemical separation methods, as discussed in chapter 2.3.

#### 2.2.2. Terbium-152

Currently, the most efficient technique for generating terbium-152 is through the high-energy proton-induced spallation of tantalum targets, similar to the process employed for terbium-149, resulting in yields of a few hundreds of MBq at CERN-ISOLDE 27. The improvements foreseen with ISOL@MYRRHA and IMPACT-TATTOOS are expected to further improve the purity and yields for terbium-152.

**Figure 4:**
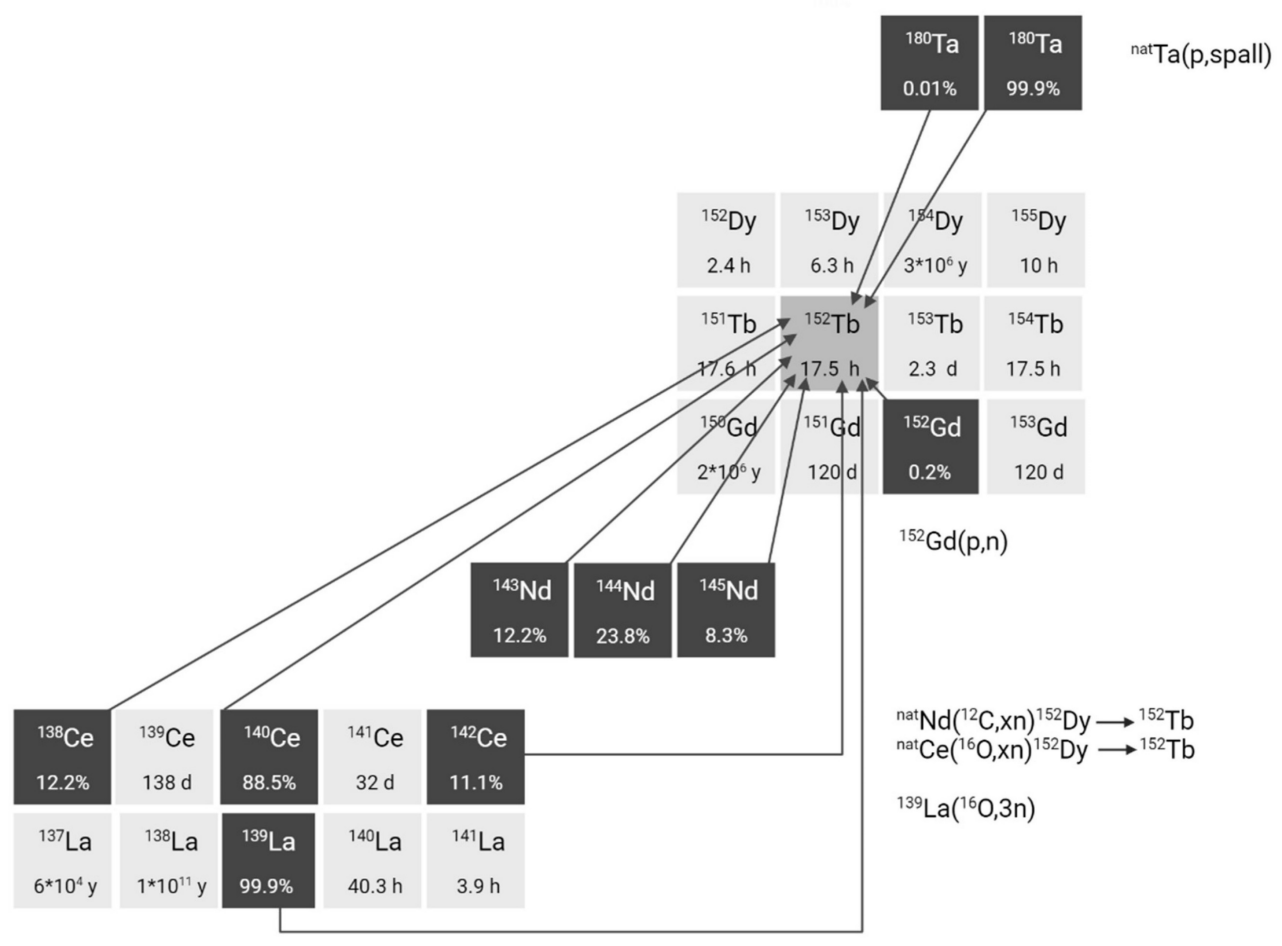
Section of the nuclide chart illustrating possible production routes of terbium-152 26. Created with BioRender.com.

Although alternative approaches have been explored, achieving the required radionuclide purity for terbium-152 production remains challenging. One example involves heavy ion induced reactions such as ^nat^Nd(^12^C,xn)^152^Dy(ɛ)^152^Tb, ^139^La(^16^O,3n)^152^Tb, and ^nat^Ce(^12^O,xn)^152^Dy(ɛ)^152^Tb (**Figure 4**) 28-30. However, the two indirect reactions have the disadvantage of producing dysprosium-150, dysprosium-151, and dysprosium-153 in addition to dysprosium-152. Consequently, the produced terbium-152 will always be contaminated with dysprosium isotopes and, especially, their daughter products terbium-150, terbium-151, and terbium-153 28,29. Similarly the direct ^139^La(^16^O,3n)^152^Tb reaction results in the production of a significant amount of terbium-151 in addition to terbium-152 30. In addition, the relatively low cross-sections result in an overall low yield of terbium-152. To address these challenges, further optimization is required encompassing factors such as beam energy and target thickness. Furthermore, the development of highly efficient separation techniques is crucial to obtain radionuclidic pure terbium radionuclides. Interestingly, a number of studies have explored the possibility of producing terbium-152 by irradiation of gadolinium targets through ^152^Gd(p,n)^152^Tb 31-34. Due to the low content of gadolinium-152 (0.2%) in natural gadolinium, natural gadolinium targets are considered unsuitable for the production of terbium-152 for nuclear medicine 32. Köster *et al.* showed that terbium-152 can be produced with reasonable purity (<1% of terbium-153) by irradiation of highly enriched gadolinium-152 targets (99.9%) at 12 MeV 34. Moreover, based on the previously obtained data, the radionuclidic purity of terbium-152 can further be improved by reducing the proton beam energy to 10-11 MeV 34. Despite the cost of target enrichment, the method is considered one of the most promising ways to produce terbium-152 aside from proton-induced spallation.

#### 2.2.3. Terbium-155

For nuclear medicine applications, terbium-155 can be produced by irradiation of tantalum targets with high-energy proton beams combined with off-line or on-line mass separation similar to the production of terbium-149 and terbium-152 23,35. This production route results in high terbium-155 purity but suffers from pseudo-isobaric contamination of cerium-139 in the form of ^139^Ce^16^O, which needs to be removed using radiochemical purification protocols. At CERN-ISOLDE, yields of approximately 200 MBq at end of collection have been achieved, though production remains limited to a few times per year. In contrast, CERN-MEDICIS offers more frequent production opportunities, though yields are somewhat lower, typically in the range of a few tens of MBq.

**Figure 5:**
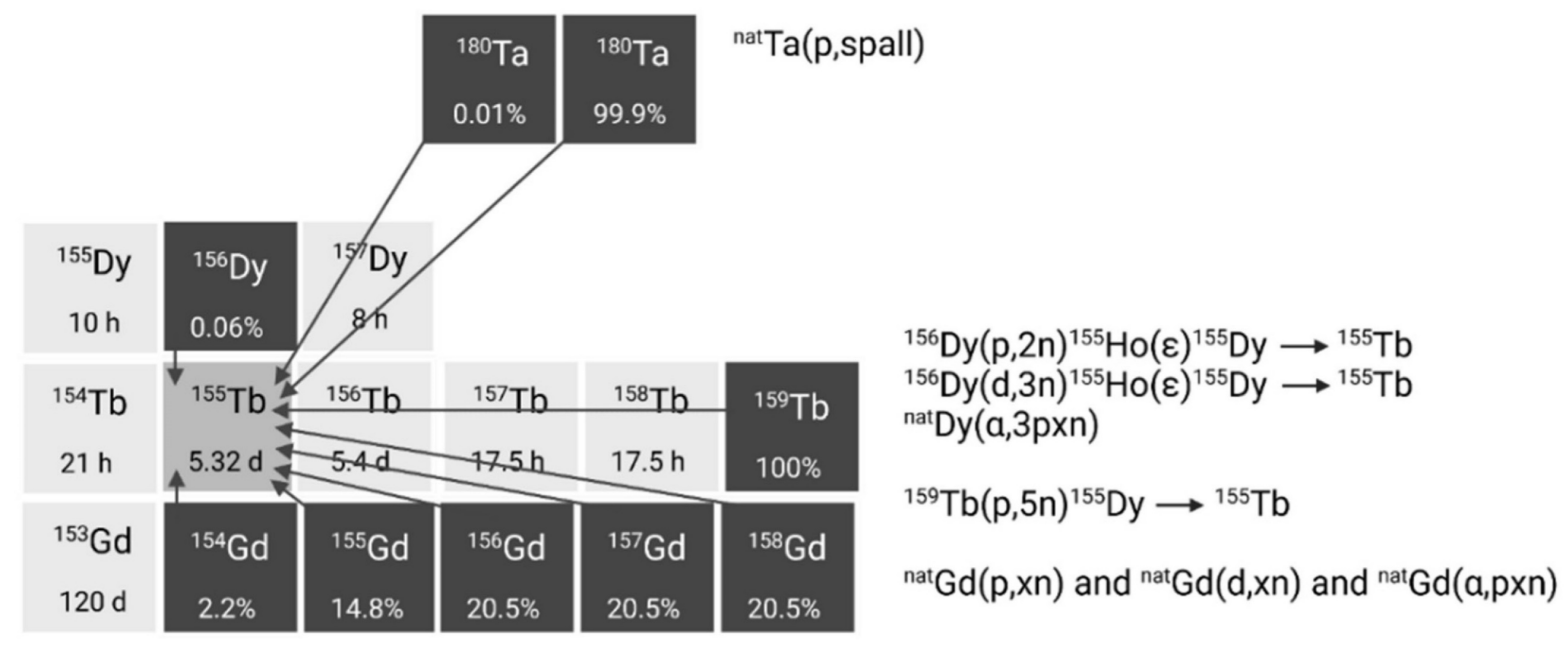
Section of the nuclide chart illustrating possible production routes of terbium-155 26. Created with BioRender.com.

Alternative production methods using commercial cyclotrons are considered such as the production of terbium-155 via various light and heavy particle induced reactions. For example, production methods where natural or enriched gadolinium targets are bombarded with α-particles, protons, or deuterons already yielded promising amounts of terbium-155, showing their applicability (**Figure 5**) 32,36,37. The main drawback of these methods is the need for highly enriched (close to 100%) gadolinium-155 targets in order to produce terbium-155 with sufficient radionuclide purity. Recently, an indirect production route via dysprosium-155 was developed by Moiseeva *et al.* enabling the production of terbium-155 with acceptable radionuclide purity without the need for highly enriched Gd targets 38. The primary impurity is terbium-153 (T_1/2_ = 2.34 days, E_β+,av_ = 547.41 keV, I_β+_ = 100%), constituting less than 5.4% of the terbium-155 activity. Though the impact is expected to be small, additional research is needed to fully understand the potential effects of this radionuclidic impurity on the body. Notably the use of enriched targets will further improve the yield of terbium-155 and reduce the impurities 38. In a similar manner, the production of terbium-155 through the irradiation of dysprosium targets with α-particles, protons, or deuterons was investigated 39-41. However, the radionuclide purity of the target isotope and/or the reaction cross sections were found insufficient for practical use in nuclear medicine. Finally, high yields of terbium-155 can also be provided through ^159^Tb(p,5n)^155^Dy(ε)^155^Tb without the need for target enrichment. Nonetheless, more powerful cyclotrons are required for the reaction and highly efficient separation methods are needed to obtain high levels of radionuclide purity 31.

#### 2.2.4. Terbium-161

Terbium-161 is mainly produced in nuclear reactors through thermal neutron irradiation of highly enriched ^160^Gd_2_O_3_ targets (typically ≥ 97.5% enriched), allowing for high production yields 42. During this process, the target undergoes neutron capture, resulting in the production of short-lived gadolinium-161 (T_1/2_ = 3.66 min) which subsequently decays into terbium-161 via β^-^ decay. Terbium-161 can then be isolated from its gadolinium-160 target matrix via radiochemical separation methods. The production route of terbium-161 is similar to that of lutetium-177 (^176^Yb(n,γ)^176^Yb → ^177g^Lu), though the cross section of the ^176^Yb(n,γ) reaction (2.8 barn) is roughly twice as large as that of the ^160^Gd(n,γ) reaction (1.5 barn) 43. Consequently, longer irradiation times and/or higher neutron fluxes are required to achieve a batch of terbium-161 with the same activity 43. Theoretically, terbium-161 can also be produced through double neutron capture (2n,γ) using a natural terbium target, which is monoisotopic in terbium-159 44,45. However, this method has several drawbacks, including reduced production yields, lower molar activity levels, and decreased radionuclidic purity. These limitations are, in part, attributed to the inability to chemically separate the newly formed terbium-161 from the terbium-159 target matrix. Additionally, the longer-lived terbium-160 (T_1/2_ = 72.3 days) remains an undesirable contaminant, making it unsuitable for application in nuclear medicine 44,45.

**Figure 6:**
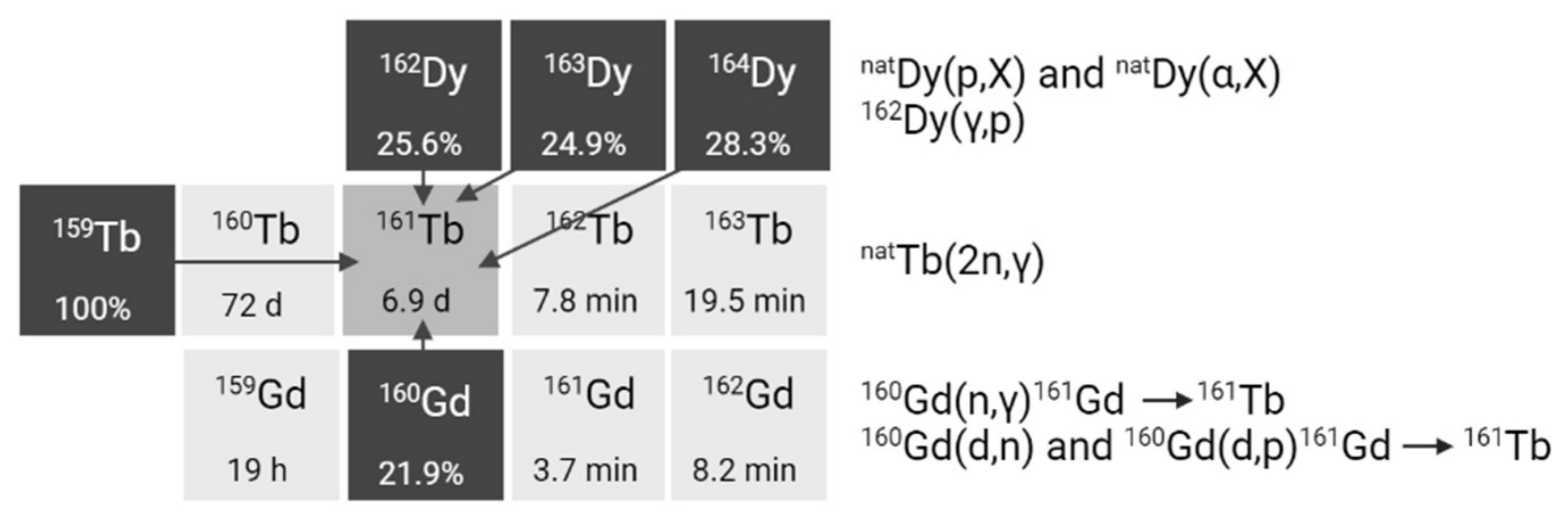
Section of the nuclide chart illustrating possible production routes of terbium-161 26. Created with BioRender.com.

Alternatively, terbium-161 can also be produced at a cyclotron by deuteron induced reactions (**Figure 6**) 43. Using this method, terbium-161 is produced via two distinct reactions: ^160^Gd(d,n)^161^Tb and ^160^Gd(d,p)^161^Gd(ε)^161^Tb with the latter predominating. It should be noted that even by using highly enriched gadolinium-160 (100%), the production of a substantial amount of contaminating terbium-160 (T_1/2_ = 72.3 days) is inevitable. Hence, the cyclotron method cannot compete with the reactor method with regards to purity and production yield 43. Few other methods have also been explored for the production of terbium-161, such as ^nat^Dy(p,X) and^ 162^Dy(γ,p) 46,47. Nevertheless, they were found ineffective for the production of terbium-161 for nuclear medicine due to the low production cross sections and presence of the isotopic impurity terbium-160 46,47.

To conclude, production of terbium-149 with sufficient radionuclide purity relies on high energy spallation coupled with mass separation carried out exclusively at specialized production facilities. This method is also preferred for the production of terbium-152 and terbium-155, though these radionuclides may also be produced in cyclotrons by irradiation of highly enriched gadolinium targets. The practical implementation of these methods is, however, hindered by the requirement of highly enriched targets. A promising alternative for producing terbium-155 is the proton irradiation of terbium-159 targets, which eliminates the need for highly enriched targets as natural terbium is monoisotopic in terbium-159. Nonetheless, the co-production of other terbium isotopes might still require the need for mass separation to obtain sufficient purity of the terbium isotope of interest. High yields of terbium-161 can be relatively easily achieved in nuclear research reactors with high thermal neutron flux irradiating highly enriched gadolinium-160 targets, with the possibility of generating terabecquerel amounts of terbium-161 with high (radio-)chemical and high radionuclidic purity. Therefore, the potential of upscaling terbium-161 production has matured over the last decade, reminiscent of the large-scale manufacturing of lutetium-177 by commercial entities. Nonetheless, a strategic approach to the enrichment of gadolinium is critical to ensure this potential.

### 2.3. Chemical separation methods for carrier-free terbium radionuclides

For applications in nuclear medicine, carrier-free radionuclides with high radionuclidic purity and high (radio-)chemical purity are required. To meet this requirement, radiochemical separation methods are employed to separate the desired terbium radionuclides from the irradiated target matrix as well as other radiocontaminants formed during irradiation. These methods typically involve chromatographic methods to separate terbium from its decay products and isobaric impurities. Here, we present a concise overview of the most relevant separation methods for obtaining no-carrier-added terbium radionuclides.

As mentioned previously, terbium-149, terbium-152, and terbium-155 are mainly produced using proton-induced spallation on tantalum targets coupled with mass separation to obtain high radionuclidic purity of the desired radionuclide. However, further purification is required as mass separation alone is unable to differentiate between isobaric and pseudo-isobaric species. This need for additional purification becomes particularly evident in the case of terbium-149. Given that terbium-149 has a relatively short half-life, comparable to the time of its collection, terbium-149 is prone to contamination by its decay products, namely, gadolinium-149 and europium-149 as well as pseudo-isobaric ions, ^133^Ce^16^O^+^ and ^133^La^16^O^+^ 48,49. To mitigate this contamination, chemical separation is usually performed using cation exchange chromatography with an aqueous carboxylic acid solution as the eluent, or making use of a combination of LaNthanide (LN)-type resins achieving separation by using various concentrations of HNO_3_. The same method is also employed to separate terbium-152 from isobaric impurities. Similarly, chemical separation of terbium-155 from pseudo-isobaric ^139^Ce^16^O species is required to obtain radiologically pure terbium-155 35. In this regard, selective oxidation of interfering ions (Ce^2+^ and Ce^4+^) is necessary before chromatographic separation to ensure the purity of terbium radionuclides. For instance, sodium bromate can be used to selectively oxidize Ce^2+^ and Ce^4+^ after which extraction chromatography using 8 M HNO_3_ can be used to elute terbium-155 without contaminating cerium 35. With off-line mass separation, pre-separation by means of thermo-chromatography can be used, whereby the cerium oxide is released at a lower temperature than the terbium isotopes, providing a higher purity sample 50.

The production of no-carrier-added terbium-161 involves its isolation from the irradiated gadolinium target. Cation exchange chromatography methods are used to provide [^161^Tb]TbCl_3_ in dilute hydrochloric acid solution, mirroring the process of commercially available lutetium-177 42,51. Alternatively, Aziz and Artha reported the quantitative separation of terbium-161 from gadolinium targets using extraction chromatography on an LN resin column and sequential elution of gadolinium in 0.8 M HNO_3_ solution and terbium in 3 M HNO_3_ 52. More recently an automated lab-scale method was developed that relies on small solid-phase extraction using a combination of LN-type extraction resins, making it more compatible with automation using commercially available systems like the Trasis All-in-One system 53. Using this technique, terbium-161 could be successfully purified from smaller scale targets with high radionuclide and chemical purity. Though the researchers have suggested that there is room for further improvements to minimize the presence of the gadolinium-160 target material and the dysprosium-161 decay product in the final terbium-161 sample, it remains evident that full automation of the purification process holds immense potential when it comes to upscaling production and managing larger quantities of target material 53. These small modular systems could be interesting for final refinement of terbium-161 after bulk separation on a different location or system, or could be used for fast reprocessing of terbium-161 after several days of decay to remove the dysprosium-161 daughter reducing its interference during radiolabeling.

## 3. Coordination Chemistry of Terbium

Terbium is a silver-white rare earth-metal first discovered in 1843 by Carl Gustaf Mosander 54. The natural fluorescence and magnetic properties of terbium have made the element attractive for commercial applications. For example, terbium is used in color TV tubes, fluorescent lamps, and electronic devices 54. For radiopharmaceutical applications, the chemical properties of terbium play a significant role in the selection of the most optimal chelator for radiolabeling. Hence, this chapter focusses on the fundamental chemistry of terbium.

As a member of the lanthanide series, terbium has 65 electrons which are arranged in an [Xe]4f^9^6s^2^ electron configuration. Like other lanthanide elements, terbium is predominantly found in the +3 oxidation state as a result of the 6s and 5d valence electrons 55,56. Throughout the lanthanide series the 4f subshell is filled with electrons which are tightly bound due to the high effective nuclear charge and do not participate in bond formations. Due to the increasing nuclear charge and poor shielding of the 5s and 5p electrons by the 4f subshell, the ionic radius contracts when moving from lanthanum (1.16 Å) to lutetium (0.977 Å), hence the term “lanthanide contraction”. As a result, the charge density and hydration energy (-ΔH_hydr_) increases with increasing atomic number, affecting the coordination number (CN) of the trivalent lanthanide ion. In general, the CN of cationic aqua complexes [Ln(H_2_O)_x_]^3+^ is assumed to be 9 for early lanthanides (i.e. La-Eu) and 8 for late lanthanides (i.e. Dy-Lu), while intermediate lanthanides, such as Tb, form a mixture of 8- and 9-coordinate species. Moreover, Tb^3+^ is classified as a hard Lewis acid with a high positive charge and small ionic radius, i.e. high charge density, and is strongly hydrated in aqueous media. Hence, chelators for Tb^3+^ need to be designed accordingly. As hard Lewis acids preferably coordinate with hard Lewis bases comprising electron-donating oxygen (e.g. carboxylates) and nitrogen (e.g. amines) donor atoms, multidentate ligands with a suitable cavity comprising these donor atoms can yield thermodynamically stable complexes, though they might require some additional energy (i.e. heat) to overcome the high hydration energy of the Tb^3+^ ion 55,56.

## 4. Bifunctional Chelating Ligands

In radiometal-based radiopharmaceuticals, bifunctional chelators are used to ensure a stable connection between the radiometal and the vector molecule. These chelators contain two crucial components: a metal complexing agent and a functional group that forms a covalent bond with the vector molecule (e.g. isothiocyanate or an active ester). For an ideal chelator, it is essential to enable fast and quantitative radiolabeling of vector molecules under conditions that are compatible with the vector molecule. In addition, the radiometal complex should exhibit high thermodynamic stability and kinetic inertness to prevent *in vivo* dissociation through transchelation with serum proteins or competing metal ions. As mentioned previously, the stability of the complex is determined by the chemical properties of the radiometal, including charge, preference of donor ligand and coordination chemistry, making it a crucial factor to consider when selecting a chelator. The subsequent paragraphs will provide an overview of the most important chelators currently used for complexation of terbium radionuclides.

### 4.1. Acyclic chelators: DTPA and DTPA-Derivatives

**Figure 7:**
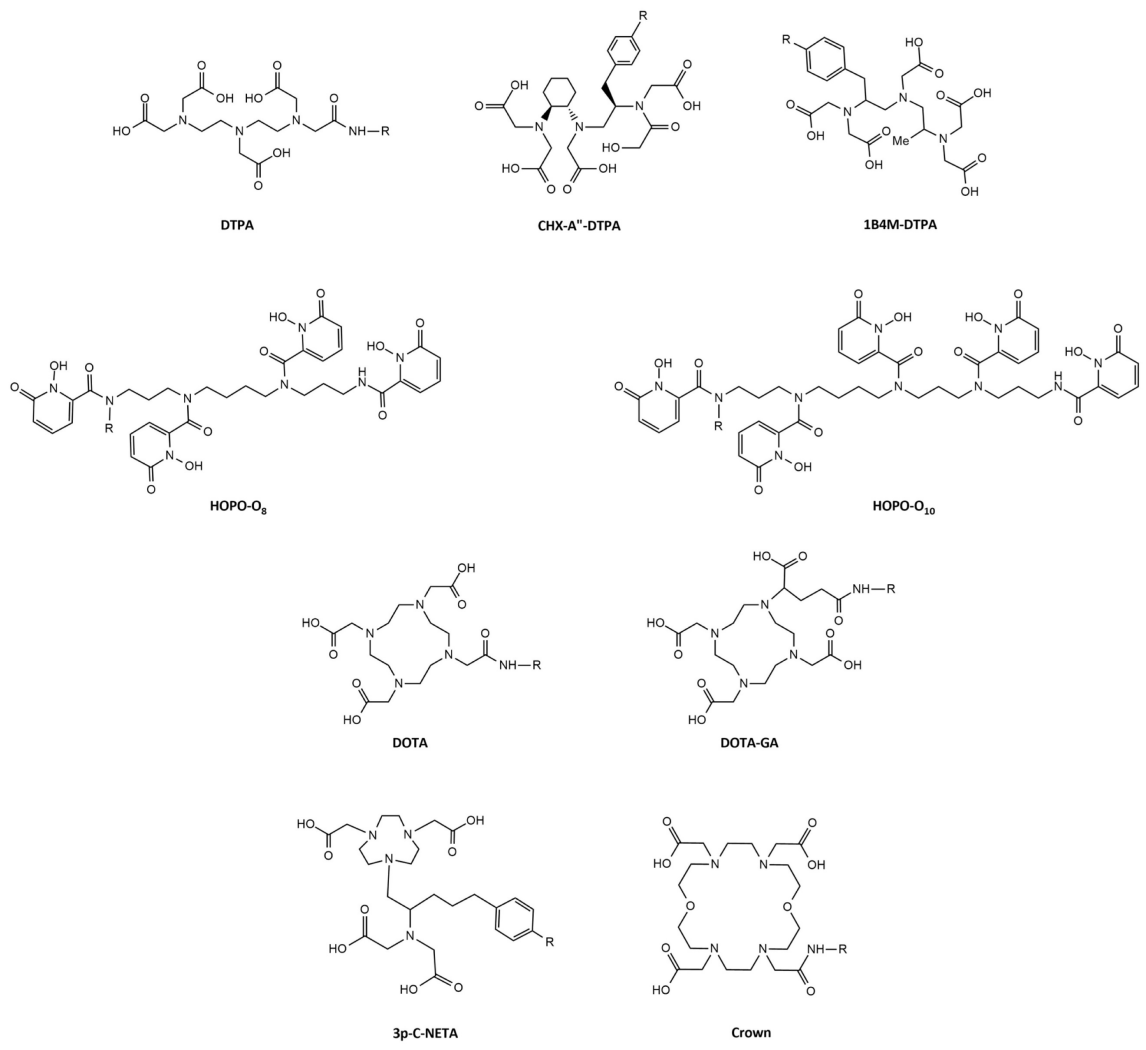
Chemical structures of bifunctional chelators used for complexation of terbium radionuclides.

Diethylenetriaminepentaacetic acid (DTPA) is one of the oldest chelators (**Figure 7**). Its flexible acyclic nature allows rapid radiolabeling of several radiometal ions at room temperature, including Tb^3+^ (logK_ML_ = 22.71) 57. However, due to the flexible structure of DTPA, the resulting complexes exhibit only moderate *in vivo* stability. Consequently, DTPA has been largely replaced by more advanced chelators such as modified DTPA derivatives, including: 2-(4-isothiocyanatobenzyl)-6-methyldiethylene-triaminepentaacetic acid (1B4M-DTPA) and [(*R*)-2-amino-3-(4-isothiocyanatophenyl)propyl]-*trans*-(*S*,*S*)-cyclohexane-1,2-diamine-pentaacetic acid (CHX-A”-DTPA) (**Figure 7**) 58,59. 1B4M-DTPA was synthesized through the addition of a methyl substituent onto one of the ethylene backbones of DTPA which introduces steric hindrance, effectively impeding the release of a radiometal from the chelator 58,59. As a result, the derivative has been used for the FDA-approved ^90^Y-based radiotherapeutic Zevalin® for treatment of non-Hodgkin's lymphoma 60. Conversely, CHX-A”-DTPA has a more rigid structure due to its cyclohexyl backbone which imposes a partially pre-organized metal ion binding site 58. This modification increases the kinetic inertness of the complex, though longer reaction times are required for coordination compared to DTPA 58. Since its introduction, the use of CHX-A”-DTPA has been extensively investigated for several radiometals such as indium-111, yttrium-86/90, and lutetium-177 61-67. However, research efforts with terbium radionuclides have been somewhat limited. Nonetheless, a recent study by Cassells *et al*. has investigated the potential of CHX-A”-DTPA in radiolabeling heat-sensitive biomolecules with terbium radionuclides 68. The study underscores the exceptional efficiency of CHX-A”-DTPA in radiolabeling (i.e. radiochemical yield >98% at 25 °C), even at low ligand concentrations. However, despite its efficiency, CHX-A”-DTPA proved to be a suboptimal chelator for coordinating Tb^3+^ ions due to its poor *in vitro* and *in vivo* stability, resulting in significant off-target bone uptake and retention 68.

Aside from commercially available chelators, researchers have explored the unique chemical properties of the radionuclides to rationally design highly specific chelators. One relevant example is the use of HOPO-based bifunctional chelators: 3,4,3-LI(1,2-HOPO), referred to as HOPO-O_8_, and 3,4,3,3-(LI-1,2-HOPO), referred to as HOPO-O_10_. Compared to the octadentate HOPO-O_8_ chelator, HOPO-O_10_ has an extended pentaamine backbone allowing the incorporation of an extra 1,2-HOPO moiety. This structural modification increases the number of oxygen atoms available for complexation allowing decadentate complexes 69. Recently, a preliminary study demonstrated the superiority of both chelators for the complexation of [^155^Tb]Tb^3+^ and [^161^Tb]Tb^3+^ at room temperature compared to the gold-standard DOTA (described in section 4.2.). Though, research on the application of HOPO chelating agents for the complexation of terbium radionuclides is rarely reported, these promising *in vitro* findings pave the way for further investigation into their potential within the field of nuclear medicine 69.

### 4.2. Macrocyclic chelators: DOTA and DOTA-Derivatives

The macrocyclic 1,4,7,10-tetraazacyclododecanetetraacetic acid (DOTA) chelator and its derivatives have been widely applied in the radiopharmaceutical field (**Figure 7**) 58. The rigid macrocyclic structure allows the formation of complexes with high thermodynamic stability and kinetic inertness. In particular, DOTA forms highly stable bonds with trivalent lanthanide ions, such as Tb^3+^ (logK_ML_ = 24.2), through coordination with the hard oxygen atoms from its four carboxylic groups, while the nitrogen atoms within the macrocyclic ring provide additional stabilization to the metal center 58,70. However, it's worth noting that coordination with DOTA typically requires longer reaction times and higher temperatures (30-60 min at 95°C) compared to linear chelators. This can be problematic when working with short-lived radionuclides and heat-sensitive biomolecules. Moreover, studies have demonstrated that achieving quantitative yields with DOTA often demands the use of high ligand concentrations 68,71. Despite its disadvantages, DOTA remains the most utilized chelator for complexation of medical isotopes like actinium-225, bismuth-213, copper-64, galium-68, lutetium-177, and terbium-161 and finds clinical applications in compounds such as [^225^Ac]Ac-DOTATOC, [^64^Cu]Cu-DOTATATE, [^68^Ga]Ga-DOTATATE, [^68^Ga]Ga-DOTATOC, [^177^Lu]Lu-DOTATATE (Lutathera®), and [^177^Lu]Lu-PSMA-617 (Pluvicto^TM^) 72-77. To overcome some of its limitations, researchers have explored various modifications to the DOTA framework, introducing additional binding tethers to enhance its kinetics. One such modification is DOTA-GA (GA = glutaric acid) which contains an additional free carboxylate group compared to DOTA (**Figure 7**). Due to this modification, DOTA-GA has demonstrated faster complexation kinetics and improved stability with hard acids such as gallium-68 and lutetium-177 78,79. Notably, the additional negative charge provided by the DOTA-GA chelator plays an important role in stabilizing the positive charges of the trivalent metal core 56,79. Furthermore, the introduction of an additional carboxylate group in DOTA-GA brings unique advantages for labelling lanthanides as it allows the use of the extra carboxylate group for efficient conjugation with the vector molecule 56.

Recently, a promising macrocyclic chelator has been developed to allow efficient radiolabeling of large metal ions, known as 2,2',2'',2'''(1,10-dioxa-4,7,13,16-tetraazacyclooctadecane-4,7,13,16-tetrayl)-tetraacetic acid (crown) (**Figure 7**) 80. Although, the chelator was originally designed for actinium-225 complexation, it is currently being evaluated for its effectiveness in complexing terbium radionuclides. Preliminary findings have highlighted its capacity to efficiently form stable complexes with terbium-155 and terbium-161, achieving quantitative yields under mild conditions (room temperature, 10-minute reaction time, and pH of 6.0) 81.

### 4.3. Hybrid chelators: NETA and NETA-Derivatives

In hybrid chelators the acyclic and macrocyclic characters are combined to integrate the advantages of both frameworks, providing rapid complexation kinetics at mild temperatures (= acyclic framework) and prolonged stability (= macrocyclic framework) 82. An example of such a chelator is {4-[2-(biscarboxymethylamino)-5(4-nitrophenyl)-entyl]-7-carboxymethyl-[1,4,7]triazonan-1-yl}acetic acid (3p-*C*-NETA) which combines a more rigid 1,4,7-triazacyclononane-*N,N'*-diacetic acid (NODA) backbone with a flexible acyclic tridentate pendant arm (**Figure 7**). Notably, 3p-*C*-NETA has been reported to be a promising chelator for radiolanthanides yttrium-90 and lutetium-177 based on its fast coordination kinetics and improved stability 83. A recent study by Ahenkorah *et al.* further elucidated its capabilities by evaluating its radiolabeling properties with a variety of radionuclides, including lutetium-177, bismuth-213, copper-67, and terbium-161 (**Figure 7**) 84. The results were encouraging, with excellent quantitative yields (> 95%) achieved at moderate temperatures (< 55°C). [^177^Lu]Lu- and [^161^Tb]Tb-3p-*C*-NETA complexes showed excellent *in vitro* stability in both PBS and human serum for a minimum of 24 h, while [^67^Cu]Cu- and [^68^Ga]Ga-3p-*C*-NETA complexes were stable in PBS for over 24 h, but not in human serum 84. Moreover, a comprehensive evaluation and comparison of DOTA, DOTA-GA, and 3p-*C*-NETA chelators for radiolabeling heat-sensitive compounds with terbium radionuclides was performed, demonstrating good radiochemical conversion (RCC >90%) for DOTA, DOTA-GA, and 3p-*C*-NETA-constructs at a concentration of 10 µM and moderate temperatures of 40°C 68. Interestingly, the RCC decreased when lower concentrations were used for DOTA and DOTA-GA, but remained quantitative (>95%) for 3p-C-NETA-constructs, even at a concentration of 0.1 µM. Moreover, the resulting ^161^Tb-labeled 3p-*C*-NETA complexes displayed remarkable stability *in vitro* and *in vivo* 68. These findings collectively suggest that 3p-*C*-NETA may serve as a more suitable chelator for the complexation of Tb^3+^ ions at mild temperatures, compared to rigid chelators such as DOTA and DOTA-GA. Furthermore, the stability of the complexes formed with 3p-*C*-NETA surpasses that of the terbium complex obtained with the acyclic chelator CHX-A”-DTPA.

## 5. Preclinical and Clinical Proof-of-concept Studies with Terbium-based Radiopharmaceuticals

An overview of preclinical and clinical studies with terbium radionuclides is provided in **Table 2**.

### 5.1. Terbium-149

In 2004, Beyer *et al.* reported the first preclinical study with terbium-149. In the study, disease progression was evaluated in a mouse model of leukemia following targeted radionuclide therapy (TRT) with ^149^Tb-labeled rituximab, a CD20-targeting antibody 85. In this study, mice were injected with [^149^Tb]Tb-CHX-DTPA-rituximab (1.11 GBq/mg, 5 μg) two days after tumor cell inoculation, before the development of manifested tumors. The treatment demonstrated remarkable efficacy, leading to tumor-free survival of 89% of the treated mice over a period of 4 months, without detectable signs of toxicity. Additionally, a preliminary dose estimation for patients injected with [^149^Tb]Tb-CHX-DTPA-rituximab revealed that a therapeutic administered activity of 5 GBq would lead to a radiation exposure to the bone marrow of 540 mSv, which is significantly lower than the often cited critical level of 2 Sv for this dose limiting organ 85.

**Figure 8:**
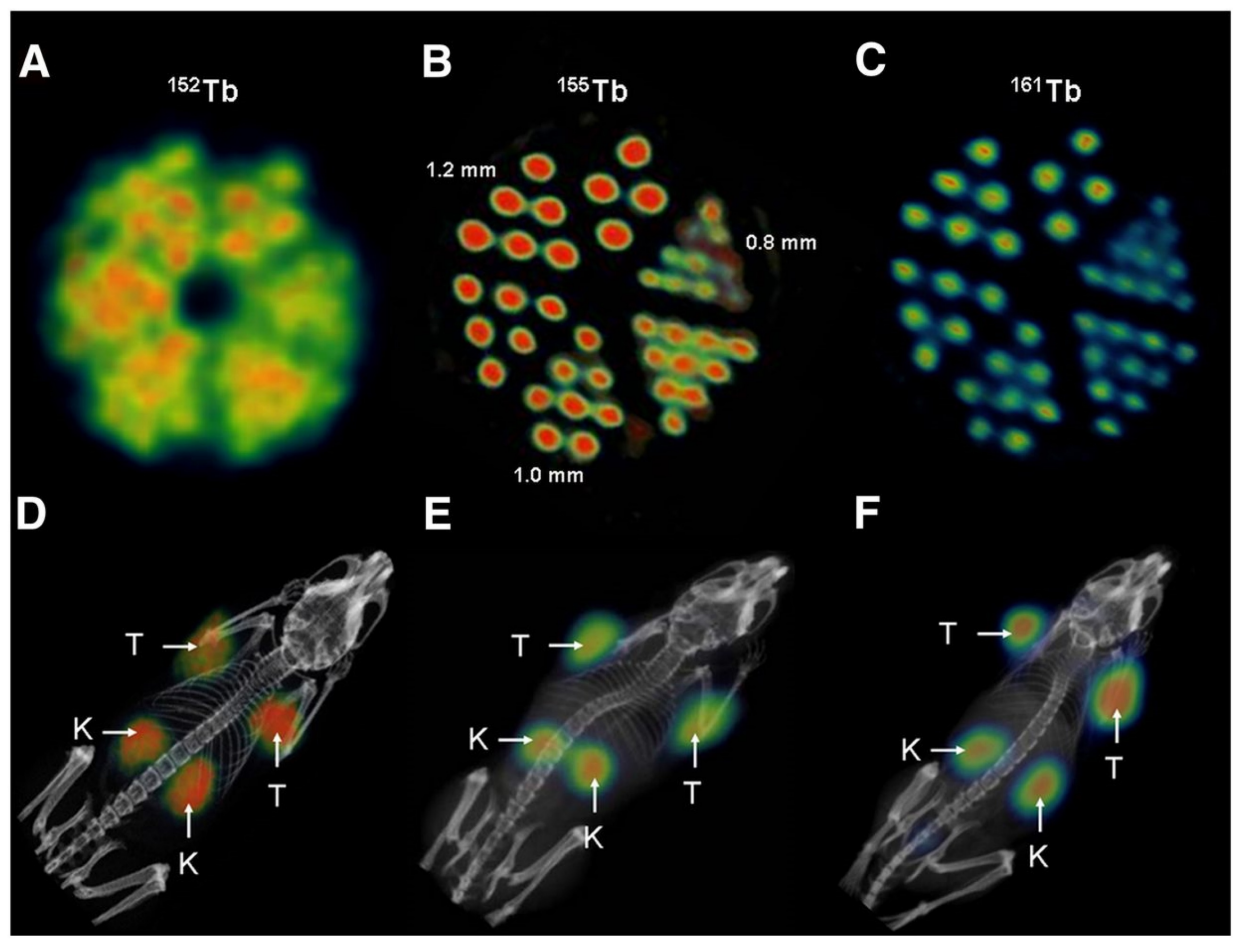
(A) PET image of Derenzo phantoms of terbium-152 (1.9 MBq). (B-C) SPECT images of Derenzo phantoms of terbium-155 (0.6 MBq) and terbium-161 (50 MBq), respectively. (D) PET/CT image of KB tumor-bearing mouse at 24 h after injection of [^152^Tb]Tb-cm09, (E-F) SPECT/CT images of KB tumor-bearing mice at 24 h after injection of [^155^Tb]Tb-cm09 and [^161^Tb]Tb-cm09, respectively. (K = kidney; T = KB tumor xenograft). Reprinted with permission from Müller* et al.* 49, copyright 2012 by the Society of Nuclear Medicine and Molecular Imaging, Inc.

TRT with terbium-149 was further investigated in combination with a DOTA-folate conjugate (cm09) in a folate receptor-positive tumor mouse model 49. Interestingly, this landmark study was the first proof-of-concept study performed with all four terbium radionuclides demonstrating their applicability for PET (terbium-152) and SPECT (terbium-155) imaging as well as for targeted α- (terbium-149) and β^-^-/Auger electron- (terbium-161) therapy 49. Specifically, radionuclide therapy using [^149^Tb]Tb-DOTA-cm09 and [^161^Tb]Tb-DOTA-cm09 was found to inhibit tumor growth in mice, resulting in significantly longer survival times compared to untreated controls. Furthermore, [^152^Tb]Tb-DOTA-cm09 and [^155^Tb]Tb-DOTA-cm09 allowed for excellent visualization of the tumor lesions in the mice (**Figure 8**). Based on these results, it is likely that terbium-152 and terbium-155 could serve as suitable diagnostic alternatives to terbium-149 and terbium-161, which would enable accurate diagnosis, dosimetry, and therapy monitoring.

This preclinical pilot study was later complemented by a more in-depth investigation of the antitumor efficacy of the ^149^Tb-labeled DOTA-cm09 conjugate 86. Tumor-bearing mice were injected with two different activity levels: 2.2 MBq (group A) and 3.0 MBq (group B), corresponding to an absorbed dose of ~19 Gy (group A) and ~26 Gy (group B). The TRT was effective and well-tolerated, revealing a dose-dependent effect with regards to tumor growth inhibition (A: 62%, B: 85%) and survival time (A: 30.5 days, B: 43 days), compared to the untreated control group (21 days). Additionally, an *in vitro* cell viability assay was performed, confirming a folate receptor-specific and activity-dependent therapeutic effect of the ^149^Tb-labeled DOTA-folate conjugate 86.

**Figure 9:**
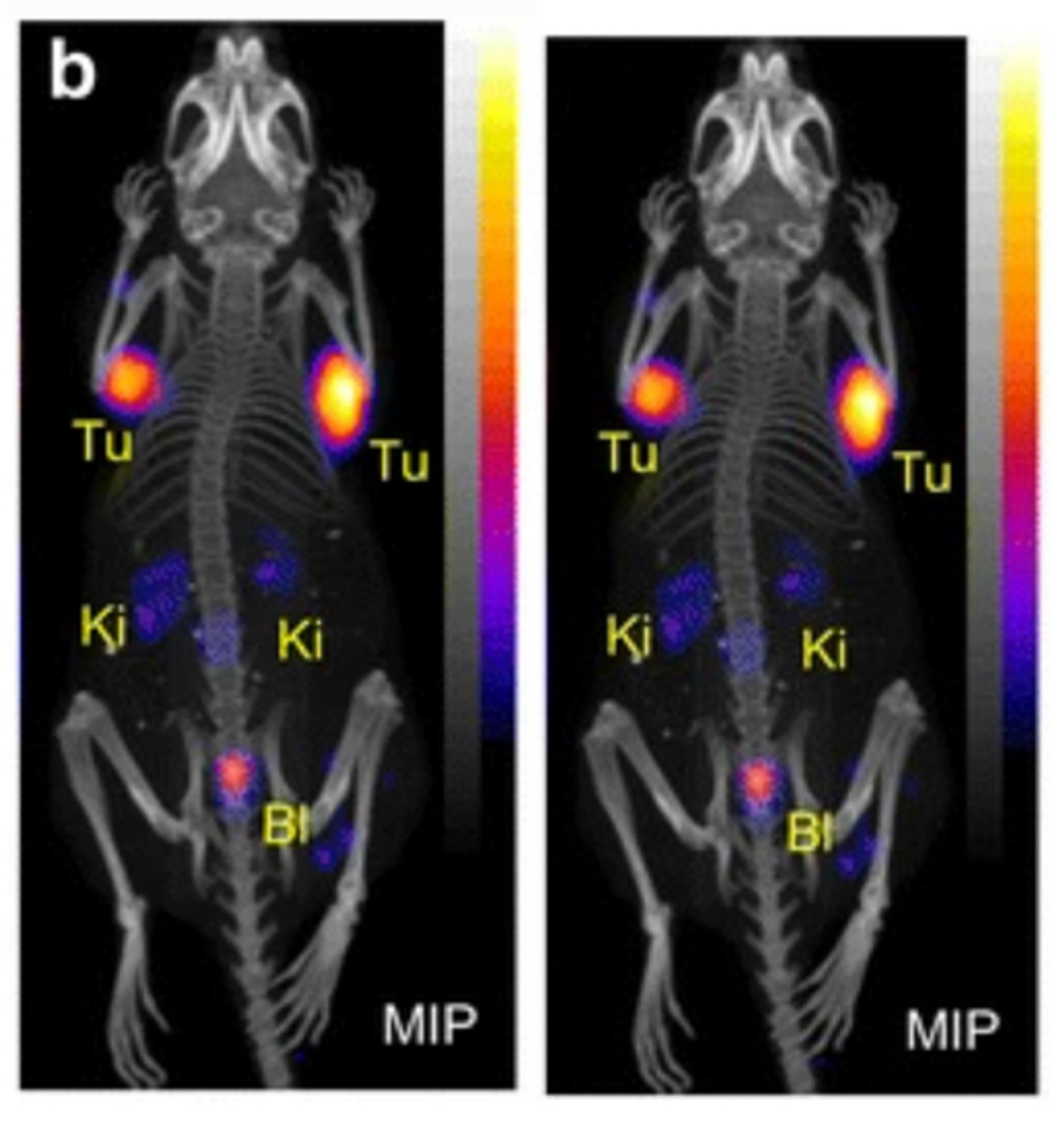
PET/CT images of an AR42J tumor-bearing mouse 2 h after injection of [^149^Tb]Tb-DOTANOC (7 MBq). (Ki = kidney, MIP = Maximal intensity projections, Tu = tumor). Reprinted with permission from Müller *et al.* 11, copyright 2016 (http://creativecommons.org/licenses/by/4.0/).

Remarkably, a ground-breaking study recently demonstrated the potential of combining α-therapy with PET using ^149^Tb-labeled DOTANOC, a clinically established somatostatin analogue 11. In this study, AR42J tumor-bearing mouse were injected with [^149^Tb]Tb-DOTANOC (7 MBq, 1.4 nmol). The high-quality PET/CT images enabled clear visualization of the tumors and specific tumor cross-sections displayed a uniform distribution of the radiopharmaceutical (**Figure 9**) 11. The ability of terbium-149 to be used for PET imaging offers the unique advantage of combining α-therapy with PET imaging using a single radionuclide, making it a promising candidate for theranostic α-radionuclide therapy, though more studies are warranted to explore its suitability in a clinical setting.

### 5.2. Terbium-152

The enhanced production yields at CERN-ISOLDE over the last decades have opened up new possibilities for more detailed preclinical studies with terbium-152. The diagnostic potential of terbium-152 was demonstrated by Müller *et al.* when used with the peptide DOTANOC in AR42J tumor-bearing mice 87. The distribution data obtained from the preclinical PET/CT scans showed that [^152^Tb]Tb-DOTANOC performed similarly to [^177^Lu]Lu-DOTANOC when used in the same animal model, indicating that terbium-152 is a promising diagnostic match for therapeutic radiolanthanides 87.

**Figure 10:**
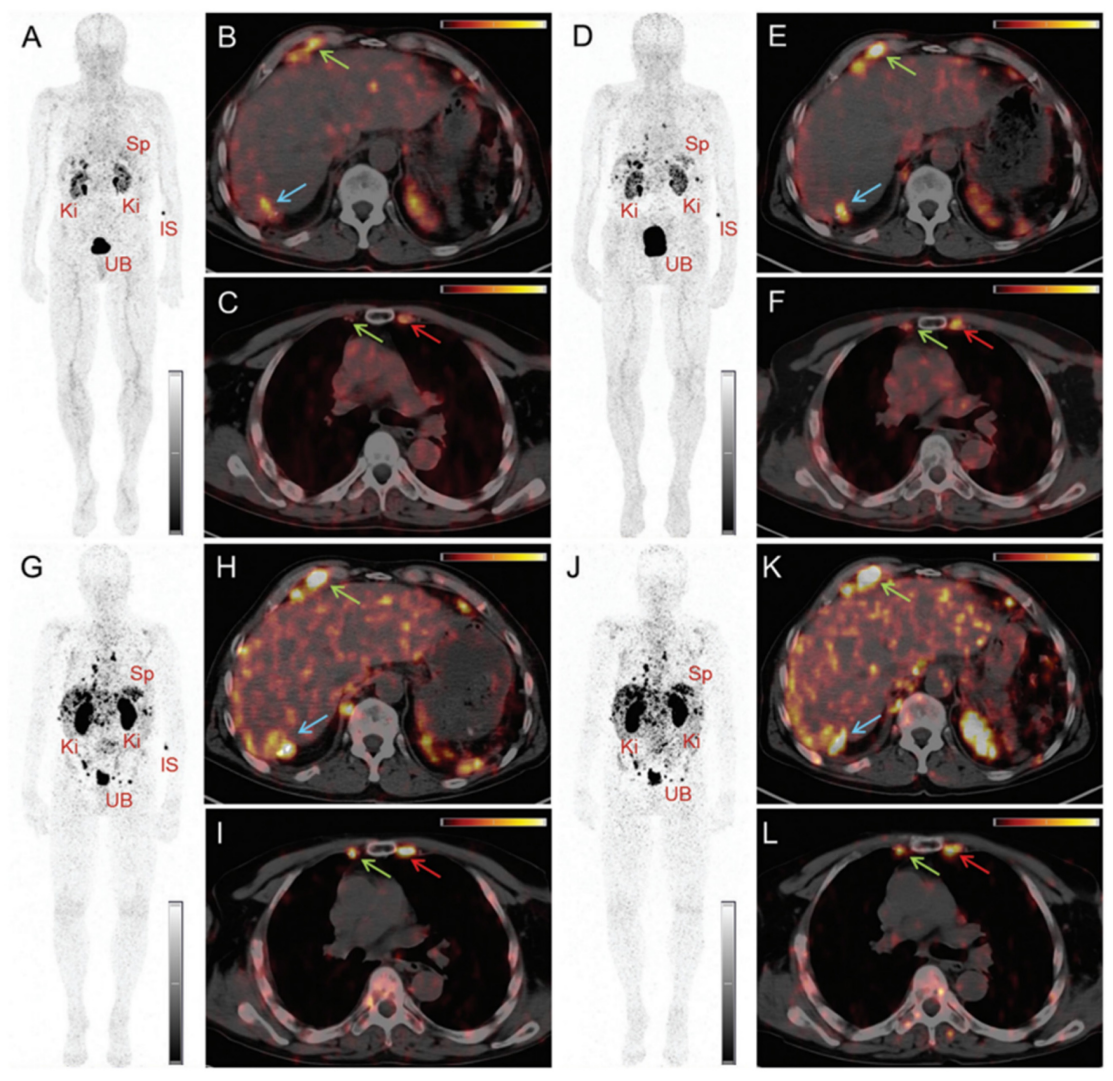
PET/CT images of a patient with neuroendocrine neoplasm of the terminal ileum obtained at 25 min (A-C), 2 h (D-F), 17 h (G-I) and 24 h (J-L) after injection of 145 MBq [^152^Tb]Tb-DOTATOC, respectively. (IS = site of injection, Ki = kidneys, Sp = spleen, UB = urinary bladder). Reprinted with permission from Baum *et al*. 88, copyright by The Royal Society of Chemistry 2017.

**Figure 11:**
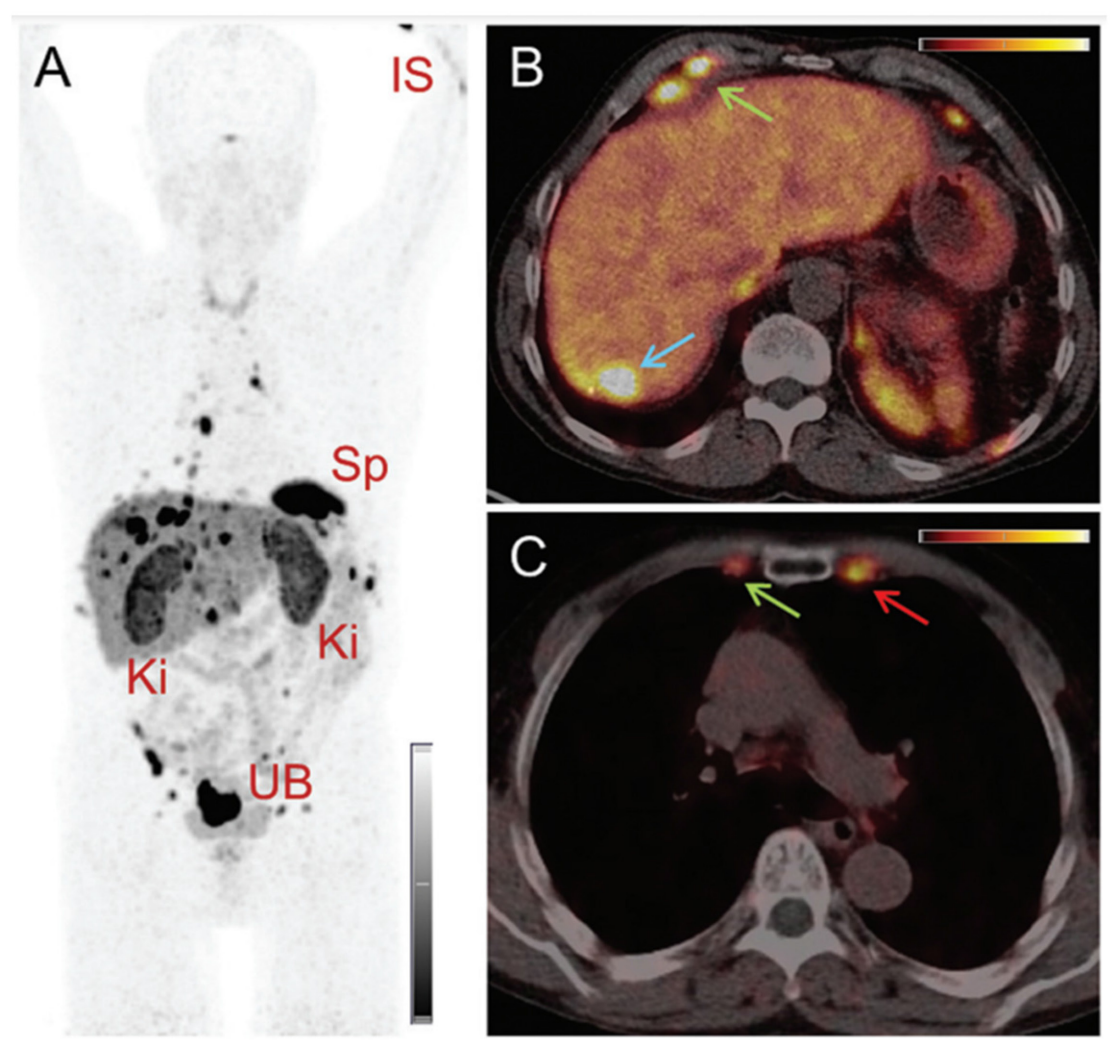
PET/CT images of the same patient acquired 55 min after injection of [ ^68^Ga]Ga-DOTATOC (104 MBq). (IS = site of injection, Ki = kidneys, Sp = spleen, UB = urinary bladder). Reprinted with permission from Baum *et al*. 88, copyright by The Royal Society of Chemistry 2017.

Moreover, the clinical feasibility of [^152^Tb]Tb-DOTATOC for PET/CT imaging was further investigated in a patient with a metastatic neuroendocrine neoplasm 88. The study's findings showed the effectiveness of [^152^Tb]Tb-DOTATOC (145 MBq, 2.5 MBq/kg) in visualizing small metastases, though the image quality was found to be noisier compared to PET images obtained using [^68^Ga]Ga-DOTATOC (104 MBq) (**Figure 10-11**). Nonetheless, the PET/CT scan using [^152^Tb]Tb-DOTATOC effectively detected all the metastatic lesions that had previously been identified with [^68^Ga]Ga-DOTATOC, confirming its diagnostic potential 88. Additionally, the clinical potential of terbium-152 was also evaluated using a PSMA-targeting agent, PSMA-617, which is currently used in combination with lutetium-177 ([^177^Lu]Lu-PSMA-617 (Pluvicto^TM^)) for the treatment of metastasized castration-resistant prostate cancer (mCRPC) 77. The study revealed that [^152^Tb]Tb-PSMA-617 and [^177^Lu]Lu-PSMA-617 exhibited similar pharmacokinetic profiles in tumor-bearing mice, which was consistent with previous *in vitro* findings 89. Encouraged by these promising preclinical results, the researchers conducted the first-in-human application of [^152^Tb]Tb-PSMA-617 for PET/CT imaging in a patient with mCRPC (administered activity: 140 MBq). The imaging procedure was well-tolerated by the patient, and the obtained images facilitated the detection of all the metastatic lesions that were previously identified by [^68^Ga]Ga-PSMA-11 PET/CT scans 89. While terbium-152 holds promise for PET/CT imaging in patients for pre-therapeutic dosimetry and treatment planning, the superior quality of images obtained with widely-available gallium-68 raises questions about the clinical applicability and usefulness of terbium-152.

### 5.3. Terbium-155

**Figure 12:**
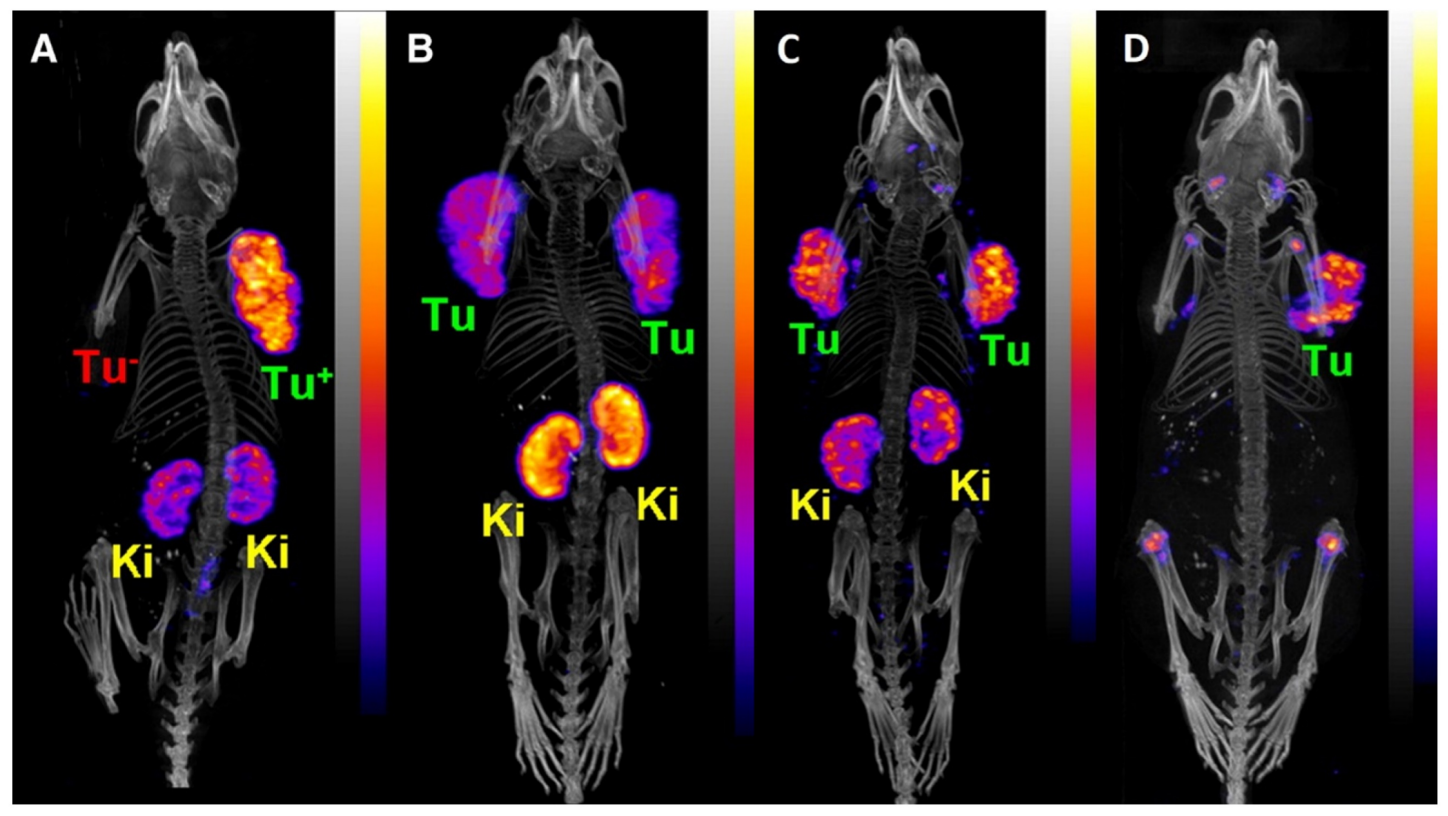
PECT/CT images of tumor-bearing mice 4 h after injection of [^155^Tb]Tb-labelled targeting vectors: (A) [^155^Tb]Tb-MD (8.0 MBq), (B) [^155^Tb]Tb-DOTATATE (6.7 MBq), (C) [ ^155^Tb]Tb-cm09 (8.5 MBq), and (D) [^155^Tb]Tb-chCE7 (4.2 MBq). (Ki = kidney, Tu = tumor). Adapted and reprinted with permission from Müller *et al*. 90, copyright © 2014 Elsevier Inc.

**Figure 13:**
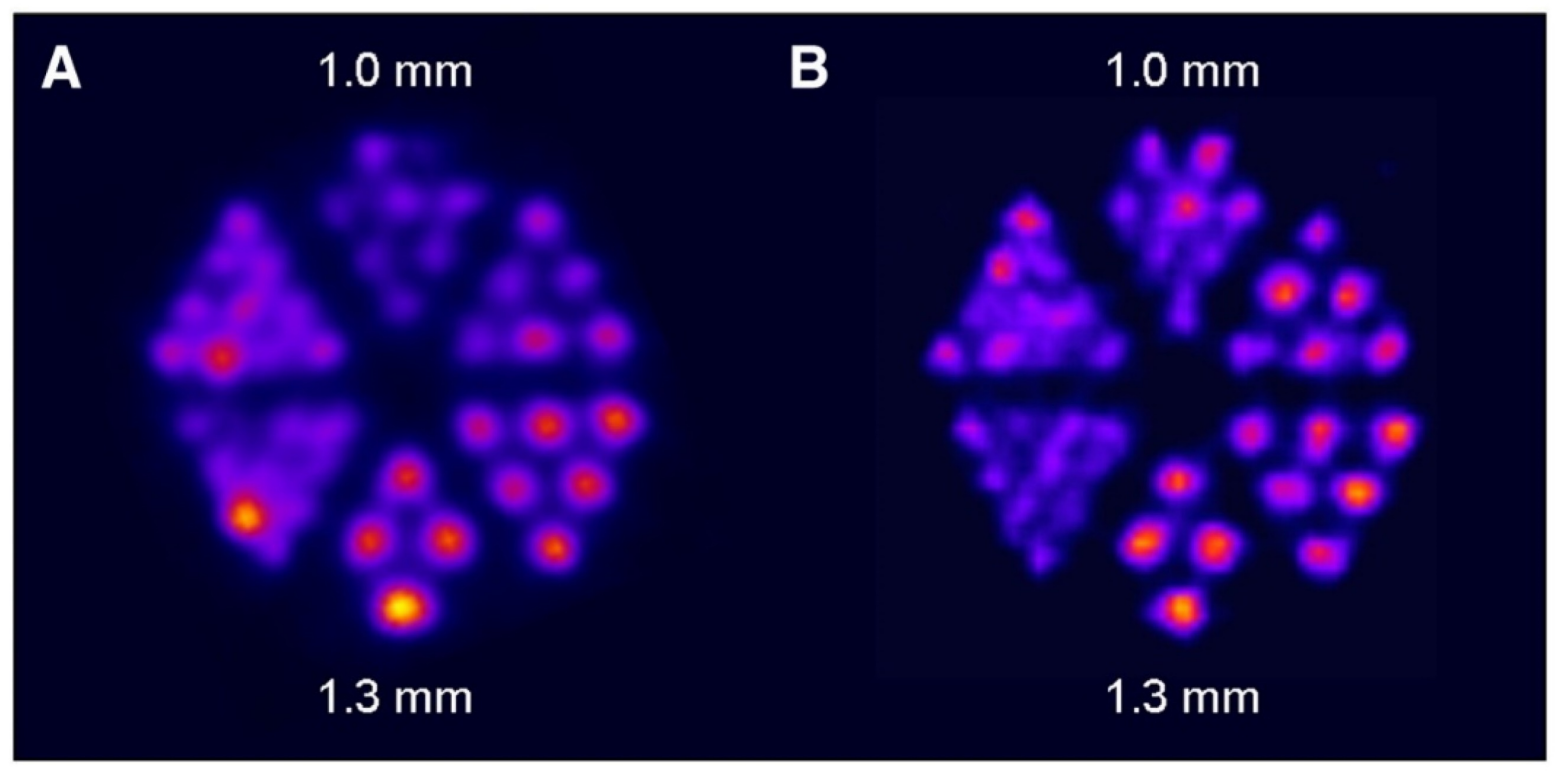
SPECT images of Derenzo phantoms filled with (A) terbium-155 (2.6 MBq) and (B) indium-111 (4 MBq). Reprinted with permission from Müller *et al*. 90, copyright © 2014 Elsevier Inc.

Following the initial proof-of-concept study of Müller *et al.* with the DOTA-folate conjugate (see 5.1.; **Figure 8**), the researchers conducted a subsequent study to further evaluate the clinical potential of terbium-155 for SPECT/CT imaging 90. Four different tumor targeting vectors of variable physiological half-lives were radiolabeled with terbium-155 and evaluated in tumor-bearing mice, including two fast-clearing DOTA-peptides: DOTATATE, a somatostatin analogue, and a minigastrin analogue (MD). Additionally, two longer-circulating vectors were investigated: a folate derivative (cm09), and chCE7, an anti-L1-cell adhesion molecule (L1-CAM) antibody. SPECT/CT scans were performed 4 h p.i. of the radiolabeled peptides, and 2 and 3 days p.i. of [^155^Tb]Tb-cm09 and [^155^Tb]Tb-CHX-A”DTPA-chCE7, respectively. The high quality images allowed clear visualization of the tumor lesions (**Figure 12**). Furthermore, µSPECT phantom studies with terbium-155 displayed excellent spatial resolution comparable to the well-established indium-111 (**Figure 13**). These promising results establish terbium-155 as a promising alternative to indium-111, particularly for pre-therapeutic dosimetry involving radiometals like yttrium-90, lutetium-177, and terbium-161 90. Notably, the absence of any additional β^+^/ β^-^ emission in terbium-155 makes it particularly favorable for diagnostic purposes, as it offers the potential to minimize patient exposure to high radiation doses, ensuring safer and more effective imaging procedures.

### 5.4. Terbium-161

In 1995, De Jong *et al.* conducted the first preclinical study involving [^161^Tb]Tb-DTPA-octreotide. They found that [^161^Tb]Tb-DTPA-octreotide exhibited promising results for TRT due to its specific uptake in somatostatin receptor-positive tissues and its high tissue/blood ratio in tumor-bearing rats 91. In recent years, there has been significant progress in the investigation of the therapeutic potential of terbium-161, leading to comparisons with the clinically-used lutetium-177.

Both lutetium-177 (T_1/2_ = 6.65 days, Eβ_av_ = 134 keV, E_γ_ = 113 keV (I = 6.17%), E_γ_ = 208 keV (I = 10.36%)) and terbium-161 have similar decay properties. However, terbium-161 is believed to have a superior antitumor effect due to the co-emission of a substantial amount of IC and Auger electrons, which can result in a more effective treatment especially with regard to microscopic metastases. Detailed dose calculations have already demonstrated a significant increase in absorbed dose through the emission of these low-energy electrons, even in micro-metastasis 16,17,19. For example, research conducted by Hindié and colleagues revealed that the dose delivered by terbium-161 is 1.8-fold higher compared to lutetium-177 in a 100 µm micro-metastasis with doses of 44.5 Gy and 24.5 Gy, respectively 19. Similarly, in a 10 µm cell, the dose from terbium-161 was found to be 3.6-fold higher, namely 14.1 Gy for terbium-161 compared to 3.9 Gy for lutetium-177. Note that these calculations were based on the assumption that 1 MeV is released per µm^3^. Furthermore, the relative contribution of the IC and Auger electrons to the absorbed energy were calculated. For lutetium-177, IC and Auger electrons accounted for 10% of the absorbed energy in a 10 mm sphere and 33.9% in a 10 µm sphere. Conversely, for terbium-161 the contribution of these low-energy electrons was found to be much higher, namely, 24.9% in a 10 mm sphere and 88.3% in a 10 µm sphere 19.

The superior therapeutic efficacy of terbium-161 has also been demonstrated in numerous *in vitro* studies and *in vivo* experiments with tumor-bearing mice. For example, the same administered activity of [^161^Tb]Tb-DOTA-cm09 proved to be more potent in reducing tumor cell viability than [^177^Lu]Lu-DOTA-cm09 in folate receptor-positive KB and IGROV-1 cell lines 13,49. Additionally, [^161^Tb]Tb-DOTA-cm09 reduced tumor growth and improved the survival time of tumor-bearing mice more efficiently 13. Similar results were obtained for [^161^Tb]Tb-PSMA-617 using PSMA-positive PC-3 PIP tumor cells, revealing that [^161^Tb]Tb-PSMA-617 was up to 3-fold more effective than [^177^Lu]Lu-PSMA-617 *in vitro* 14. Treatment of PC-3 PIP tumor-bearing mice showed an activity-dependent tumor growth inhibition and prolonged survival of mice which was more enhanced for [^161^Tb]Tb-PSMA-617 compared to [^177^Lu]Lu-PSMA-617 14. The superior therapeutic efficacy of terbium-161 over lutetium-177 was further confirmed in combination with the anti-L1CAM antibody chCE7, PSMA-I&T, and the albumin-binding *S*-isomer of Ibu-DAB-PSMA (SibuDAB) 93,94.

Contrary to prior assumptions suggesting that internalization is a crucial factor for the efficacy of short-ranged IC and Auger electrons, the non-internalizing somatostatin antagonist [^161^Tb]Tb-DOTA-LM3 has shown to be substantially more effective than its ^177^Lu-based counterpart in inhibiting *in vitro* tumor cell viability, being up to 100 times more potent. In comparison, the internalizing somatostatin agonist [^161^Tb]Tb-DOTATOC was only 5-fold more effective in inhibiting tumor cell viability than the ^177^Lu-counterpart. This result was further confirmed *in vivo*, revealing that the membrane localizing [^161^Tb]Tb-DOTA-LM3 was significantly more effective in delaying tumor growth than [^177^Lu]Lu-DOTA-LM3 and [^161^Tb]Tb-DOTATOC 15. This discovery has prompted a paradigm shift in our understanding of the Auger electron effect, stimulating ongoing research efforts to elucidate the membrane-related effects of these short-range electrons 5,98.

In conclusion, these preclinical data, supported by dosimetric calculations, undoubtedly indicate that ^161^Tb-labeled radiopharmaceuticals hold clear conceptual advantages over their ^177^Lu-labeled counterparts for tumor therapy. Clinical trials exploring the potential of ^161^Tb-labeled radiopharmaceuticals in a clinical setting will be critical in solidifying the clinical utility of ^161^Tb-based therapies.

**Figure 14:**
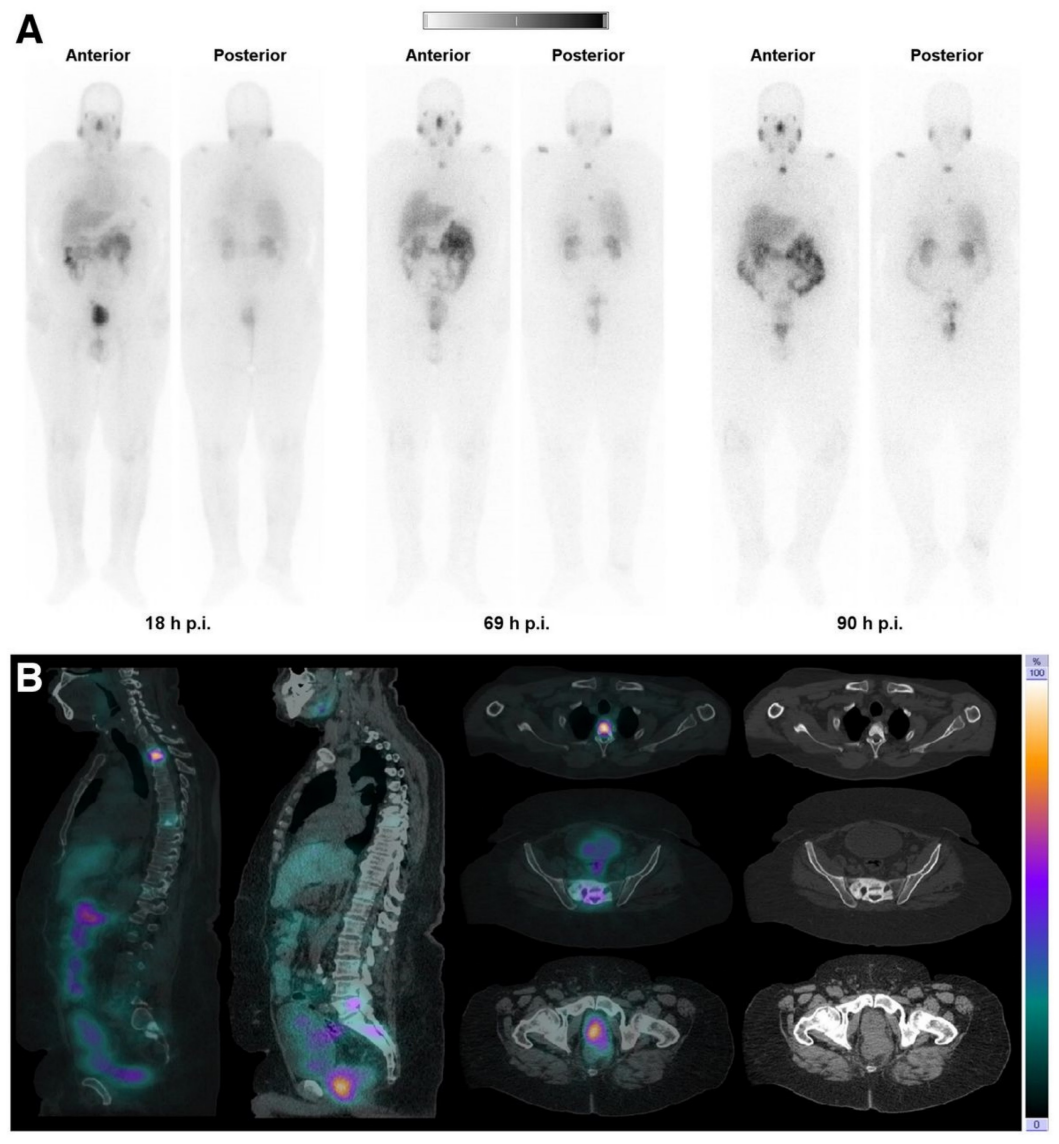
(A) Whole-body images at different time points after injection of 5550 MBq of [^161^Tb]Tb-PSMA-617. (B) Representative SPECT/CT sagittal and axial slices and CT axial slices demonstrating physiologic biodistribution in lacrimal, parotid, and submandibular glands; nasopharyngeal mucosa; liver; intestinal tract; kidneys; and urinary bladder, as well as pathologic uptake in primary prostate tumor and metastatic bone lesions. (p.i. = post-injection). Reprinted with permission from Al-Ibraheem *et al*. 96, copyright 2023 by the Society of Nuclear Medicine and Molecular Imaging.

The feasibility of SPECT imaging through the γ-ray emission of terbium-161 has also been thoroughly investigated 13,15,81,93. Interestingly, the first-in-human SPECT/CT imaging using [^161^Tb]Tb-DOTATOC and [^161^Tb]Tb-PSMA-617 showed encouraging results as the obtained images were of good quality and enabled visualization of all the previously identified tumor lesions using [^68^Ga]Ga-DOTATOC (**Figure 14**) 95,96. Dual-isotope SPECT imaging with terbium-161 and lutetium-177 also becomes possible due to the difference in γ-energies, as demonstrated in preclinical studies 15,94. The simultaneous visualization of these two distinct isotopes also provided further evidence confirming the previously hypothesized interchangeability of these radionuclides without any significant impact on their tissue biodistribution 15,94. The promising outcomes of this research suggest that future (pre)clinical investigations involving terbium-161 can confidently rely on the preclinical data acquired from its ^177^Lu-labeled counterpart. This would accelerate research efforts, allowing researchers to directly focus on exploring the therapeutic efficacy of terbium-161, which appears to exhibit superior effects compared to lutetium-177 15.

Notably, only a limited number of studies have addressed the potential side effects of terbium-161 radionuclide therapy on normal tissues, despite kidney and bone marrow toxicity being well-known risks of radionuclide therapy. In this context, an important study conducted by Haller *et al.* investigated the long-term effects of [^161^Tb]Tb-DOTA-cm09 treatment on the kidneys 92. The functional evaluation of the kidneys and histopathological analysis revealed no additional damage to the kidneys as compared to equal activities of [^177^Lu]Lu-DOTA-cm09. Hence, Auger electrons are unlikely to result in additional renal damage which is in line with previous observations regarding Auger electron-based therapy with [^111^In]In-octreotide in patients 92,99. The reason for the lack of additional renal toxicity was attributed to the limited tissue range of Auger electrons 99. According to this hypothesis, these low-energy electrons would be unable to cause damage to the radiosensitive glomeruli. Instead, any radiation dose would be confined to the more radioresistant tubular cells, where the radioconjugates tend to accumulate, without detrimental physiological effect 99.

Currently, several clinical trials are ongoing, exploring the potential of ^161^Tb-labeled radiopharmaceuticals in a clinical setting. One noteworthy study has just commenced with the aim to measure the therapeutic index of [^161^Tb]Tb-DOTA-LM3 in comparison to the current standard [^177^Lu]Lu-DOTATOC in patients with gastroenteropancreatic neuroendocrine tumors (NCT05359146). In addition, ongoing research is dedicated to assessing the safety and efficacy of two promising radiopharmaceuticals [^161^Tb]Tb-PSMA-I&T and [^161^Tb]Tb-PSMA-617 as part of the VIOLET (NCT05521412) and the REALITY trial (NCT04833517), respectively. Interestingly, an insightful case study has recently been published, offering initial evidence of the clinical therapeutic potential of [^161^Tb]Tb-PSMA-617 97. The study involved an 85-year-old man with advanced mCRPC and disease progression after eight cycles of [^177^Lu]Lu-PSMA-617 radionuclide therapy. He received one cycle of 6.5 GBq [^161^Tb]Tb-PSMA-617, and after four weeks, a partial remission was documented with a notable decrease in plasma tumor marker (prostate-specific antigen, PSA) and tumor burden which was visualized with PET/CT imaging 97. As comprehensive data on the extended efficacy of ^161^Tb-based radionuclide therapy is not yet available, the results of these clinical trials are highly anticipated. Meanwhile, the ongoing clinical studies and promising preliminary evidence continue to support the therapeutic potential of ^161^Tb-based radiopharmaceuticals.

## 6. Prospects

The terbium quadruplet, comprising terbium-149, terbium-152, terbium-155, and terbium-161, holds immense potential in nuclear medicine. At the moment, several vector molecules are under investigation for their clinical potential in combination with terbium radionuclides including, somatostatin analogues (DOTATOC, DOTANOC and DOTATATE), prostate-specific membrane antigen ligands (PSMA-617 and PSMA-I&T), and cancer targeting antibodies (rituximab and chCE7). Interestingly, since the same element is used, the vector molecule can be radiolabeled with different terbium radionuclides using identical protocols and without changing pharmacokinetic properties. This unique characteristic makes terbium ideal for the “matched-pair” principle of theranostics.

Diagnostic terbium radionuclides might be of particular interest for pre-therapeutic dosimetry and treatment planning 89. Their relatively long half-life enables imaging at later time points post-injection which would allow for higher tumor-to-background ratios compared to images obtained with its gallium-68 (T_1/2_ = 68 min) counterpart, as demonstrated in a clinical study with [^152^Tb]Tb-PSMA-617. Nevertheless, gallium-68 provides PET images with a superior quality compared to terbium-152 at early timepoints which raises questions about the clinical utility of terbium-152 as gallium-68 is already well-established in the clinic and widely available. The relatively long half-life of the diagnostic terbium isotopes also poses certain drawbacks, primarily leading to increased radiation doses delivered to healthy tissues. It's worth noting, however, that these radiation dose concerns are insignificant in the context of pre-therapeutic applications. In this instance, the additional absorbed dose would account for only a very small fraction of the dose delivered by the therapeutic counterpart 89.

Out of the four terbium radionuclides, terbium-161 is the most advanced in terms of production, preclinical and clinical evaluation. Due to its similarity to the clinically-established lutetium-177, along with the beneficial co-emission of low energy IC and Auger electrons, terbium-161 has emerged as a highly attractive radionuclide for therapeutic applications. Indeed, the superior therapeutic efficacy of terbium-161 over lutetium-177 has been demonstrated in numerous preclinical studies supporting dosimetric calculations. Though it was first believed that the highest relative biological effectiveness (RBE) of Auger electron emitters results when these radionuclides localize in highly radiosensitive organelles such as the nucleus, research has indicated that the cell membrane and mitochondria might also be good targets. This discovery has prompted a paradigm shift in our understanding of the Auger electron effect, stimulating ongoing research efforts to further elucidate the radiobiological effects of membrane- and mitochondria-targeting Auger electron emitters. Nevertheless, the increased therapeutic effect at lower doses could minimize undesired side-effects, such as hematological and renal toxicity. At the moment, several clinical trials are ongoing to assess the safety and therapeutic efficacy of terbium-161 in patients. One interesting example is [^161^Tb]Tb-DOTA-LM3, which presents a promising clinical candidate for TRT, combining the benefits of somatostatin receptor antagonists with the unique advantages of terbium-161. Encouraging results from these clinical studies could potentiate more research efforts, facilitating the clinical translation of terbium-161.

Importantly, current research efforts are also focusing on enabling precise and standardized activity measurements of terbium-161 for its clinical use. As more than 99% of the γ-rays emitted by terbium-161 have a considerable low energy (<100 keV), the geometry of the container and filling volume can significantly influence the measured activity 100-102. Therefore, to allow for precise activity measurements of terbium-161 in preclinical and clinical settings, it is necessary to use calibrated ionization chambers for standardized containers and geometries, taking into account the volume of the solution 100-102.

In comparison, research data on terbium-149, terbium-152, and terbium-155 remains limited. Terbium-152 and terbium-155 are of great interest for PET and SPECT imaging, respectively. Meanwhile terbium-149 is considered a promising radionuclide for α-therapy. However, the production of these terbium radionuclides with sufficient radionuclide purity remains challenging and is currently only possible at specialized production facilities such as ISOLDE and MEDICIS at CERN and ISAC at TRIUMF. Nonetheless, several new production facilities are under construction across the world, offering new prospects for the production of these radionuclides. Of particular concern is the short-lived terbium-149 (T_1/2_ = 4.12 h) as its use is confined to the immediate vicinity of the production site. At the moment, the limited availability of these radionuclides hinders the advancement into clinical trials and their potential clinical applicability, despite the encouraging preclinical data. The prospect of overcoming this production challenge presents a significant opportunity for more extensive preclinical investigations and clinical trials. Notably, terbium-155 could potentially be produced in medical cyclotrons with high yields. However, realizing these potential demands considerable technological advancements, as the reaction requires powerful cyclotrons and highly efficient separation methods to attain high levels of radionuclide purity.

In conclusion, the terbium quadruplet, consisting out of terbium-149, terbium-152, terbium-155, and terbium-161, holds remarkable promise in nuclear medicine. Terbium-161 is the most advanced in terms of production and (pre-)clinical evaluation, demonstrating immense potential for radionuclide therapy. Conversely, the clinical translation of terbium-149, terbium-152, and terbium-155 have been hindered by challenges in their production pathways. Overcoming these challenges in the future, could lead to more extensive (pre-)clinical investigations and their progression into clinical trials.

## Figures and Tables

**Table 1:** Decay characteristics and applications of clinically interesting terbium radionuclides. (IC = internal conversion) 7,8,9.

Radionuclide	Half-life	Decay mode (branching [%])	E_α_ [MeV]	E_β,av_ [MeV]	E_γ_ [KeV] (I_γ_ [%])	Application
^149^Tb	4.12 h	α (16.7) β^+^ (7.1) EC (76.2)	3.967	0.730	165.0 (26.4) 352.2 (29.4) 388.6 (18.4) 652.1 (16.2)	α-therapy and/or PET imaging
^152^Tb	17.5 h	β^+^ (20.3) EC (79.7)	-	1.140	271.1 (9.5) 344.3 (63.5) 586.3 (9.2) 778.9 (5.5)	PET imaging
^155^Tb	5.23 d	EC (100)	-	-	86.6 (32.0) 105.3 (25.1) 180.1 (7.5) 262.3 (5.3)	SPECT imaging
^161^Tb	6.96 d	β^-^ (100)	-	0.154	25.6 (23.2) 48.9 (17.0) 74.6 (10.3)	β^-^- and Auger/IC therapy

**Table 2:** Overview of preclinical and clinical studies with terbium radionuclides.

Radioconjugate	Vector	Procedure	Principle findings	Disease model	Reference
**Terbium-149**					
[^149^Tb]Tb-CHX-DTPA-rituximab	Antibody	IV injection of 5.5 MBq conjugate (1.1 MBq/μg) or 5-300 μg unlabeled rituximab	A significant increase in survival time (>120 days in 89% of treated mice) was found compared to the controls.	Burkitt leukemia (MM)	85
[^149^Tb]Tb-DOTA-cm09	Vitamin	IV injection of 2.2 or 3 MBq conjugate (1.2 MBq/nmol).	A significant tumor growth delay was found in treated animals; analysis of blood parameters revealed no signs of acute toxicity to the kidneys or liver in treated mice.	Cervical carcinoma (MM)	86
[^149^Tb]Tb-DOTANOC	Peptide	PET/CT scans were obtained 2 h p.i. of 7 MBq conjugate (5 MBq/nmol)	High quality PET/CT images were obtained showing distinct accumulation of radioactivity in the tumors.	Pancreatic neuroendocrine tumor (MM)	11
**Terbium-152**					
[^152^Tb]Tb-DOTANOC	Peptide	PET/CT images were obtained at different time points (2 h -22 h) p.i. of up to 47 MBq conjugate (10 MBq/nmol) or 47 MBq [^177^Lu]Lu-DOTANOC (10 MBq/nmol)	The tissue distribution profile of [^152^Tb]Tb-DOTANOC was equal to that of [^177^Lu]Lu-DOTANOC. It was also demonstrated that the molar amount of injected peptide was crucial with regard to the tumor uptake of radioactivity and tumor-to-kidney ratios.	Pancreatic neuroendocrine tumor (MM)	87
[^152^Tb]Tb-DOTATOC	Peptide	PET/CT images were obtained at 25 min and 24 h p.i. of 145 MBq conjugate (1.37 MBq/nmol)	PET/CT scans were of diagnostic quality and enabled detection of all the metastatic lesions that had previously been identified with [^68^Ga]Ga-DOTATOC.	Patient with neuroendocrine neoplasm	88
[^152^Tb]Tb-PSMA-617	Peptide	PET/CT images were obtained 2 h and 15 h p.i. of 10 MBq conjugate (10 MBq/nmol) or 25 MBq [^177^Lu]Lu-PSMA-617 (25 MBq/nmol)	The pharmacokinetic properties were found similar to those of [^177^Lu]Lu-PSMA-617.	Prostate cancer (MM)	89
		PET/CT scans were obtained at 50 min, 2 h, 18.5 h, and 25 h, p.i. of 140 MBq conjugate.	The clinical PET/CT images were of diagnostic quality, enabling the visualization of all target lesions previously detected with [^68^Ga]-PSMA-11. No adverse effects were observed or reported by the patient during, immediately after, or at follow-up checks of the patient.	Patient with mCRPC	89
**Terbium-155**					
[^155^Tb]Tb-DOTA-cm09	Vitamin	SPECT/CT images were obtained 48 h and 2 days p.i. of 8.5 MBq conjugate (5.7 MBq/nmol)	The high quality images allowed excellent visualization of the tumor lesions. SPECT phantom studies with terbium-155 displayed excellent spatial resolution comparable to the well-established indium-111	Cervical and ovarian cancer (MM)	90
[^155^Tb]Tb-DOTATATE	Peptide	SPECT/CT images were obtained 4 h p.i. of 6.7 MBq conjugate (1.8 MBq/nmol)		Pancreatic neuroendocrine tumor (MM)	
[^155^Tb]Tb-CHX-A”-DTPA -MD	Peptide	SPECT/CT images were obtained 4 h p.i. of 8.0 MBq conjugate		Epidermoid cancer (MM)	
[^155^Tb]Tb-CHX-A”-DTPA -chCE7	Antibody	SPECT/CT images were obtained 3 days p.i. of 4.2 MBq conjugate (44 MBq/mg)		Ovarian cancer (MM)	
[^155^Tb]Tb-crown-TATE	Peptide	SPECT/CT images were obtained 2.5 h p.i. of 13.8 MBq conjugate (0.71 nmol)	[^155^Tb]Tb-crown-TATE, showed high tumor targeting (32.6 %ID/g) and minimal retention in non-target organs at 2.5 h post-administration. Biodistribution studies confirmed the SPECT/CT results	Pancreatic neuroendocrine tumor (MM)	81
**Terbium-161**					
[^161^Tb]Tb-DTPA-octreotide	Peptide	IV injection of 0.2 MBq conjugate (0.4 MBq/μg)	[^161^Tb]Tb-DTPA-octreotide exhibited promising results for radiotherapy based on its specific uptake in somatostatin receptor-positive tissues and its high tissue/blood ratio.	Pancreatic neuroendocrine tumor (rat)	91
[^161^Tb]Tb-DOTA-cm09	Vitamin	Imaging: SPECT/CT images were obtained 24 h p.i. of 30 MBq conjugate (30 MBq/nmol) or 30 MBq [^177^Lu]Lu-DOTA-cm09 (30 MBq/nmol)	Imaging: SPECT/CT images confirmed equal suitability of ^161^Tb- and ^177^Lu-based conjugates for preclinical imaging purposes.	Cervical cancer (MM)	13
		Therapy: IV injection of 10 MBq conjugate (20 MBq/nmol) or [^177^Lu]Lu-DOTA-cm09 (20 MBq/nmol)	Therapy: [^161^Tb]Tb-DOTA-cm09 reduced tumor growth and improved the survival time of tumor-bearing mice more efficiently than [^177^Lu]Lu-DOTA-cm09.	Cervical cancer and ovarian cancer (MM)	13
		Renal toxicity: IV injection of 10-20-30 MBq conjugate (10 MBq/nmol) or [^177^Lu]Lu-PSMA-617 (10 MBq/nmol). Effects were investigated over 8 months	Renal toxicity: evaluation of the kidneys and histopathological analysis revealed no additional damage to the kidneys as compared to equal activities of [^177^Lu]Lu-DOTA-cm09.	Female athymic nude mice	92
[^161^Tb]Tb-PSMA-617	Peptide	IV injection of 5 - 10 MBq conjugate or [^177^Lu]Lu-DOTA-cm09	Treatment resulted in an activity-dependent increase of the median survival. Therapy with ^161^Tb-labeled conjugates was superior to [^177^Lu]Lu-PSMA-617.	Prostate cancer (MM)	14
[^161^Tb]Tb-DOTA-LM3	Peptide	IV injection of 10 MBq conjugate (50 MBq/nmol)	Tumor growth was delayed for over 44 ± 5 days and was, thus, more powerful than [^177^Lu]Lu-DOTA-LM3, which induced a tumor growth delay of only 35 ± 7 days (p<0.05).	Pancreatic neuroendocrine tumor (MM)	15
[^161^Tb]Tb -DOTA-chCE7	Antibody	IV injection of 5 MBq conjugate (600 MBq/mg) or 6 MBq [^177^Lu]Lu -DOTA-chCE7 (600 MBq/mg)	Tumor growth inhibition was better by 82.6% for ^161^Tb-DOTA-chCE7 compared to ^177^Lu-DOTA-chCE7 under equitoxic conditions.	Ovarian cancer (MM)	93
[^161^Tb]Tb-SibuDAB	Peptide	IV injection of 2, 5, or 10 MBq conjugate (1 nmol) or [^177^Lu]Lu-SibuDAB (1 nmol)	At any of the applied activities the ^161^Tb-based radiopharmaceuticals were therapeutically more effective than their ^177^Lu-labeled counterparts; [^161^Tb]Tb-SibuDAB demonstrated an approximately 4-fold higher absorbed tumor dose than [^161^Tb]Tb-PSMA-I&T due to its albumin -binding properties.	Prostate cancer (MM)	94
[^161^Tb]Tb-PSMA-I&T	Peptide	IV injection of 5 or 10 MBq conjugate (1 nmol) or [^177^Lu]Lu-PSMA-I&T (1 nmol)		Prostate cancer (MM)	94
[^161^Tb]Tb-crown-TATE	Peptide	SPECT/CT images were obtained 2.5 h p.i. of 7.95 MBq conjugate (0.7 nmol)	[^161^Tb]Tb-crown-TATE, showed high tumor targeting (30.0 %ID/g) and minimal retention in non-target organs at 2.5 h post-administration. Biodistribution studies confirmed the SPECT/CT results.	Pancreatic neuroendocrine tumor (MM)	81
[^161^Tb]Tb-DOTATOC	Peptide	SPECT/CT images were obtained at different time points p.i. of 596 MBq (patient 1) and 1,300 MBq (patient 2) conjugate	SPECT/CT images were of high quality and visualized even small metastases in bones and liver. The application of [^161^Tb]Tb-DOTATOC was well tolerated, and no related adverse events were reported.	Patient with a metastatic, well-differentiated, non-functional malignant paraganglioma (patient 1) and a patient with a metastatic, functional neuroendocrine neoplasm of the pancreatic tail (patient 2)	95
[^161^Tb]Tb-PSMA-617	Peptide	SPECT/CT images were obtained 18-69-90 h p.i. of 5,550 MBq conjugate	The obtained images were of good quality, enabling visualization of all previously identified PSMA-avid primary and metastatic bone lesions using a [^68^Ga]Ga-PSMA PET/CT scan. The application of [^161^Tb]Tb-PSMA-617 was well tolerated, and no related adverse events were reported.	Patient with mCRPC, refractory to chemotherapy.	96
[^161^Tb]Tb-PSMA-617	Peptide	Administration of one cycle of 6.5 GBq [^161^Tb]Tb-PSMA-617	After four weeks, a partial remission was documented with a notable decrease in tumor burden which was visualized with PET/CT imaging	Patient with advanced mCRPC and disease progression after eight cycles of [^177^Lu]Lu-PSMA-617 radionuclide therapy	97

Abbreviations - IV: intravenous; mCRPC: metastatic castration-resistant prostate cancer; MM: mouse model; p.i.: post-injection.

## Abbreviations

1B4M-DTPA: 2-(4-isothiocyanatobenzyl)-6-methyldiethylene-triaminepentaacetic acid
3p-*C*-NETA: {4-[2-(biscarboxymethylamino)-5(4-nitrophenyl)-entyl]-7-carboxymethyl-[1,4,7]triazonan-1-yl}acetic acid
CHX-A”-DTPA: [(*R*)-2-amino-3-(4-isothiocyanatophenyl)propyl]-*trans*-(*S*,*S*)-cyclohexane-1,2-diamine-pentaacetic acid
CN: coordination number
crown: 2,2',2'',2'''(1,10-dioxa-4,7,13,16-tetraazacyclooctadecane-4,7,13,16-tetrayl)-tetraacetic acid
CZT: cadmium zinc telluride
DOTA: 1,4,7,10-tetraazacyclododecanetetraacetic acid
DTPA: Diethylenetriaminepentaacetic acid
EC: electron capture
GA: glutaric acid
HOPO-O_10_: 3,4,3,3-(LI-1,2-HOPO)
HOPO-O_8_: 3,4,3-LI(1,2-HOPO)
IC: internal conversion
IV: intravenous
L1-CAM: anti-L1-cell adhesion molecule
mCRPC: metastasized castration-resistant prostate cancer
MM: mouse model
NODA: 1,4,7-triazacyclononane-*N,N'*-diacetic acid
p.i.: post-injection
PET: positron emission tomography (PET)
PSA: prostate-specific antigen
RBE: relative biological effectiveness
RCC: radiochemical conversion
RIB: radioactive ion beam
SPECT: single photon emission computed tomography
TRT: targeted radionuclide therapy
